# Investigation of Praziquantel/Cyclodextrin Inclusion Complexation by NMR and LC-HRMS/MS: Mechanism, Solubility, Chemical Stability, and Degradation Products

**DOI:** 10.1021/acs.molpharmaceut.1c00716

**Published:** 2021-10-21

**Authors:** Tatjana Kezele Špehar, Marijana Pocrnić, David Klarić, Branimir Bertoša, Ana Čikoš, Mario Jug, Jasna Padovan, Snježana Dragojević, Nives Galić

**Affiliations:** † Fidelta Ltd., Prilaz baruna Filipovića 29, 10 000 Zagreb, Croatia; ‡ Department of Chemistry, Faculty of Science, University of Zagreb, Horvatovac 102a, 10 000 Zagreb, Croatia; § Institute Ruđer Bošković, Bijenička cesta 54, 10 000 Zagreb, Croatia; ∥ Department of Pharmaceutical Technology, Faculty of Pharmacy and Biochemistry, University of Zagreb, A. Kovačića 1, 10 000 Zagreb, Croatia

**Keywords:** praziquantel, cyclodextrins, inclusion complexes, stability studies, degradation products

## Abstract

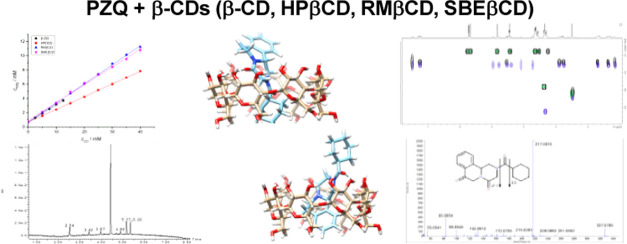

Praziquantel (PZQ) is a biopharmaceutical classification system (BCS) class II anthelmintic drug characterized by poor solubility and a bitter taste, both of which can be addressed by inclusion complexation with cyclodextrins (CD). In this work, a comprehensive investigation of praziquantel/cyclodextrin (PZQ/CD) complexes was conducted by means of UV–vis spectroscopy, spectrofluorimetry, NMR spectroscopy, liquid chromatography-high-resolution mass spectrometry (LC-HRMS/MS), and molecular modeling. Phase solubility studies revealed that among four CDs tested, the randomly methylated β-CD (RMβCD) and the sulfobutylether sodium salt β-CD (SBEβCD) resulted in the highest increase in PZQ solubility (approximately 16-fold). The formation of 1:1 inclusion complexes was confirmed by HRMS, NMR, and molecular modeling. Both cyclohexane and the central pyrazino ring, as well as an aromatic part of PZQ are included in the CD central cavity through several different binding modes, which exist simultaneously. Furthermore, the influence of CDs on PZQ stability was investigated in solution (HCl, NaOH, H_2_O_2_) and in the solid state (accelerated degradation, photostability) by ultra-high-performance liquid chromatography–diode array detection–tandem mass spectrometry (UPLC-DAD/MS). CD complexation promoted new degradation pathways of the drug. In addition to three already known PZQ degradants, seven new degradation products were identified (*m*/*z* 148, 215, 217, 301, 327, 343, and 378) and their structures were proposed based on HRMS/MS data. Solid complexes were prepared by mechanochemical activation, a solvent-free and ecologically acceptable method.

## 1 Introduction

Praziquantel (PZQ) is an anthelmintic drug widely used in developing countries to treat schistosomiasis, a neglected tropical disease caused by parasitic blood flukes of the *Schistosoma* genus.^1^ Approximately 800 million people are at risk of schistosomiasis and around 250 million are infected, with a high prevalence in the pediatric population.^2^ The fluke infection causes chronic immunogenic inflammatory, granulomatous, and fibrotic reactions, accompanied with several functional morbidities that impair normal physiological functioning and growth of an infected child.^3,4^ The global strategy to control and eradicate this disease is based on a large-scale preventive chemotherapy with PZQ, treating school-aged children mainly with a single oral drug dose of 20–40 mg/kg.^2^ PZQ is included in the World Health Organization List of Essential Medicines for treatments of adults and children as it has good efficiency, a favorable safety profile, and low production costs.^1^

The main drawbacks of PZQ include its poor water solubility, related to the high dose required, as well as intensive bitter and metallic taste, which is accompanied with poor patient compliance, especially in the pediatric population.^5,6^ Both the problems can be efficiently solved by inclusion complexation with cyclodextrins (CDs), cyclic oligosaccharides acting as multifunctional excipients.^7^ Previous studies demonstrated the ability of both natural and chemically modified CDs to improve the in vitro dissolution properties and provide efficient taste-masking through inclusion complexation.^8,9^ Furthermore, cyclodextrins can provide alternative ways of drug delivery.^10^ The enhanced solubility of the PZQ/β-CD complex, obtained by lyophilization, improved the antischistosomal efficiency of the drug in mice infected with *Schistosoma mansoni* even when administered in a subclinical dose.^11^ The complexation with β-CD enhanced the therapeutic efficiency of PZQ from 59% (for the plain drug) to 99% (for the β-CD complex), while β-CD alone had no anthelmintic activity. Therefore, the CD-based technology appears as a promising approach to develop novel and more efficient formulations for PZQ. Nevertheless, in our previous study, which aimed to develop new, solvent-free, and ecologically more acceptable methods for the preparation of PZQ/CD complexes in the solid state, we noticed an unfavorable effect of the inclusion complex formation on the chemical stability of the drug.^12^ This prompted us to thoroughly investigate the effect of CDs on the chemical stability of PZQ, as this issue has not been systematically investigated so far.

In the first part of this investigation, several phase solubility studies with PZQ and various CDs were performed to determine the exact stability constant values (*K*_s_), as data from the literature are rather inconsistent. For example, the value of *K*_s_ for PZQ complexes with β-CD varies in the range of 275–531 M^–1^.^8,9,13^ Thereafter, the complex stoichiometry was determined via liquid chromatography quadrupole time-of-flight mass spectrometry (LC-QTOF). NMR spectroscopy and molecular modeling were used to confirm the actual complex formation and gain insight into the inclusion mechanism. The second part of this investigation included solution and solid-state stability studies, focused to determine the effect of selected CDs on the stability of the PZQ, as well as characterization of observed degradation products. The results obtained in this investigation will enable a critical evaluation of the applicability of CDs in the development of novel PZQ formulations with improved functionality.

## 2 Experimental Section

### 2.1 Materials

(±) Praziquantel (PZQ) was supplied by Genera d.d. (Croatia). Cyclodextrins used in this study included β-cyclodextrin (β-CD), 2-hydroxypropyl-β-cyclodextrin (HPβCD, with an average degree of substitution, DS = 4.5), randomly methylated β-cyclodextrin (RMβCD, DS = 12), and sulfobutylether sodium salt β-cyclodextrin (SBEβCD, DS = 6.5), all obtained from CycloLab (Hungary). 1,2-Deshydro praziquantel (one of the known PZQ impurities) was obtained from Toronto Research Chemicals. Complexes in the solid state were prepared by grinding and analyzed as described previously.^12^ In addition to PZQ/CD complexes prepared in a 1:1 molar ratio, the PZQ/β-CD solid mixture was also prepared in a 1:4 molar ratio for some NMR measurements. Besides CDs and the drug, the complexes prepared did not contain any other additives.

Hydrochloric acid, min. 36.5% p.a., and ammonium bicarbonate, min. 99.5% p.a., were obtained from Sigma-Aldrich (Germany). Sodium hydroxide, pellets 2–5 mm p.a., ammonia solution, min. 25% p.a., and hydrogen peroxide, min. 30% p.a., were obtained from Kemika (Croatia). Acetonitrile and methanol, LC-MS grade, as well as acetonitrile, methanol, and isopropanol, LC grade, were obtained from Merck (Germany). Ultrapure water was obtained from a Milli-Q Advantage A10 purification system (Merck). All solvents used for NMR analysis were purchased from Eurisotop.

### 2.2 Instrumentation

UV–vis spectra were recorded on a Specord 200 spectrophotometer (Analytik Jena AG, Germany) over the range of 190–400 nm, using a standard quartz cell (*l* = 1 cm). The absorbance of PZQ was measured at 267 nm in a 1:4 methanol–water solution.

Excitation and emission spectra were recorded on a PerkinElmer LS55 spectrofluorimeter over the range of 200–400 nm using a standard quartz cell (*l* = 1 cm). The excitation and emission slit widths were 5 nm. The fluorescence intensity of PZQ was measured at 286 nm with the excitation wavelength set at 260 nm in a 1:4 methanol/water solution.

Chromatographic measurements for phase solubility were carried out on an Agilent 1220 Infinity LC, equipped with a built-in degasser, binary pump, autosampler, column oven, and variable-wavelength UV–vis detector. Chromatographic separation was achieved on a Zorbax Eclipse XDB-C18 (5 μm, 150 × 4.6 mm) column and mobile phases consisting of acetonitrile and ultrapure water in a ratio of 45:55 (v/v). The flow rate was set to 1 mL min^–1^, and the column temperature was set to ambient. The volume of injection was 10 μL, and the wavelength of detection was set to 210 nm.

High-resolution mass spectra were acquired on an Agilent 6550 Series Accurate-Mass Quadrupole Time-of-Flight (Q-TOF). Solutions were introduced directly via an Agilent 1290 Infinity II UHPLC. The electrospray ionisation mass spectrometry (ESI-MS) analysis was performed in a positive-ion mode, ranging from *m*/*z* 100 to 3200. The capillary potential was 3000 V, the fragmentor voltage was 50 V, the drying gas flow was 15 L min^–1^, and the temperature was 200 °C. The sheath gas flow was 11 L min^–1^, and the temperature was 250 °C. Nitrogen was used as a drying and sheath gas.

Accelerated solid stress testing was performed for 3 months in a Binder Constant climate chamber, model KBF 720 at 40 °C and 70% RH (relative humidity).

Photostability studies were conducted for 8 h using an ATLAS CPS+ Suntest box with a cooling aggregate, equipped with an ID65-filtered xenon lamp at 300 W m^–2^ for 1 h and 700 W m^–2^.

Chromatographic measurements for stability studies were carried out on a Waters Acquity liquid chromatography system with a UPLC diode-array detector (DAD) coupled to an Acquity SQ mass spectrometer. Chromatographic separation was achieved on a Waters Acquity BEH C18 (1.7 μm, 100 × 2.1 mm) column in gradient mode using a mobile phase consisting of 10 mM ammonium bicarbonate (NH_4_HCO_3_) pH 10 (solvent A) and acetonitrile (solvent B). The flow rate was set to 0.5 mL min^–1^, and the column temperature was set to 40 °C. The volume of injection was 10 μL. The linearity of the method was determined by injecting a series of diluted stock solutions of PZQ and PZQ-CD complexes at eight different concentrations in the range of 0.1–2.5 mg mL^–1^ (details are shown in Supporting Information (SI) Table S6). The ESI-MS spectra were obtained in a positive-ion mode ranging from *m*/*z* 50 to 1000. The source temperature was 140 °C, the desolvation temperature was 400 °C, the cone gas flow was 50 L h^–1^, and the desolvation gas flow was 750 L h^–1^.

High-resolution mass spectra of degradation products were acquired on a Sciex X500R Q-TOF System coupled with a Shimadzu Nexera X2 UHPLC System to obtain chromatographic separation using the same chromatographic conditions and column as described above. The ESI-HRMS spectra were obtained in a positive-ion mode, ranging from *m*/*z* 50 to 1000. The source temperature was 450 °C, ion source gas1 55 psi, ion source gas2 60 psi, curtain gas 30 psi, CAD gas 7 psi, collision energy 10 V, spray voltage 5500 V and declustering potential 80 V.

All NMR spectra were recorded on a Bruker Avance III 600 spectrometer equipped with an RT 5 mm inverse detection probe with *z*-gradient accessory.

### 2.3 Phase Solubility Studies

Solubility measurements were carried out according to Higuchi and Connors.^14,15^ An excess amount of PZQ was weighed, followed by an addition of 5 mL of water. Selected cyclodextrins were added into each sample to achieve a final concentration of cyclodextrin in the range of 0–40 mM (for β-CD, 0–12.5 mM). Thereafter, samples were shaken at ambient temperature for 48 h, filtered using 0.45 μm Chromafil Xtra H-PTFE filters (Macherey-Nagel, Germany), and diluted accordingly. The PZQ concentration in samples was determined by UV–vis spectrophotometry, fluorescence spectroscopy, and HPLC. Under the experimental conditions used, no interference of CDs on PZQ quantification was confirmed during method development. The apparent stability constant (*K*_1:1_) and complexation efficacy (CE) were calculated from the solubility diagram using the following formulas
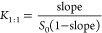

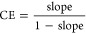
where *S*_0_ is the intrinsic solubility of PZQ. The experiments were repeated three times.

### 2.4 High-Resolution Mass Spectrometry (HRMS)—Complex Identification

Stock solutions of cyclodextrins and PZQ were prepared by weighing analytes and diluting them with water and methanol, respectively, to achieve a final concentration of 1 mg mL^–1^. Samples for HRMS analysis were prepared by diluting stock solutions in solvent, consisting of methanol and water in a ratio of 50:50 (v/v). The final concentration was 4.4 × 10^–5^ mol L^–1^. Samples of mixtures of CD and PZQ were prepared by mixing solutions in an equimolar ratio.

### 2.5 NMR Spectroscopy

PZQ (5 mg), β-CD (18.5 mg), and PZQ/β-CD complex (1:1) (23.5 mg) were dissolved in 600 μL of DMSO-*d*_6_ and transferred to a 5 mm NMR tube. The final concentration in DMSO-*d*_6_ was 16 mM.

PZQ (1.0 mg) and β-CD (2.6 mg) were each dissolved in D_2_O (1.50 and 1.08 mL, respectively) to achieve a concentration of 2.13 mM. Due to the precipitation of PZQ, it was further diluted until fully dissolved to a final concentration of 1.07 mM. To match this concentration, β-CD was also diluted. PZQ/β-CD complex (1.5 mg) was dissolved in 1.24 mL of D_2_O to a final concentration of 1.07 mM.

In an attempt to increase the concentration of PZQ in the solution, 17.09 mg of the solid mixture containing PZQ and β-CD, in a molar ratio of 1:4, was dissolved in 1 mL of D_2_O giving a final concentration of 3.52 mM for PZQ and 14.08 mM for β-CD.

All four solutions (600 mL) were transferred to 5 mm NMR tubes.

The complete NMR analysis was made on the basis of one- and two-dimensional NMR spectra (^1^H, ^13^C, COSY, NOESY, ROESY, DOSY, HSQCe, and HMBC). Unless otherwise stated, the spectra were acquired at 25 °C using standard Bruker pulse sequences. NOESY spectra were obtained with a mixing time of 500 ms. The durations of the ROESY spinlock were 200 ms (water suppression experiments) and 300 ms (recreation of the literature experiment). The data were processed using TopSpin Bruker software package.

The DOSY NMR spectra were acquired using dstebpgp3s, a pseudo-2D sequence using double-stimulated echo for convection compensation and longitudinal encode-decode (LED) with bipolar gradient pulses for diffusion and three spoil gradients.^16,17^

The spectra were acquired with 16 scans (DMSO-*d*_6_), 32 scans (β-CD in D_2_O), and 256 scans (PZQ and PZQ/β-CD complex in D_2_O) for each gradient step, and the linear gradient was chosen in the range from 2 to 95%. The diffusion times (Δ) were 93.6 ms (DMSO-*d*_6_) and 95.4 ms (D_2_O), while the gradient durations (δ) were set to 4.6 ms (DMSO-*d*_6_) and 3 ms (D_2_O). The spectra were processed using Dynamics Center 2.6.3 software. The fitted function was
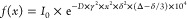
where γ is 26 752 rad/(sG), *I*_0_ is the signal integral, and variable (*x*) is the gradient strength.

### 2.6 Docking Study

The 3D model of PZQ was generated using Chem3D, while 3D coordinates of β-CD were taken from the PDB structure (PDB code: 3CGT). Docking preparation was performed with AutoDock Tools using default settings.^18^ Docking calculations were performed using AutoDock Vina 1.1.2 with docking parameters at default values.^19^ The grid spacing was 1.0, and the maximal size (126 Å × 126 Å × 126 Å) of the docking grid was used. Visualization was performed with Chimera.^20^

### 2.7 Stress Conditions

Hydrolytic degradation was carried out under acid and base conditions using hydrochloric acid (0.1 and 1 M) and sodium hydroxide (0.1 and 1 M), respectively. Hydrogen peroxide (0.3 and 3%) was used for oxidative degradation. Stock solutions of CD complexes and PZQ were prepared by weighing approximately 2 mg of PZQ and CD complexes, followed by dilution with either water (CD complexes) or acetonitrile (PZQ) to achieve a final concentration of 2 mg mL^–1^. For each condition tested, 300 μL of the stock solution was diluted with selected media to achieve a final concentration of 0.6 mg mL^–1^. All experiments were performed at 85 °C to achieve an acceptable percentage of degradation. Samples were collected at 0 min and 24 h.

### 2.8 Accelerated Solid Stress Testing

PZQ and its inclusion complexes with CDs were added into a glass screw cap vial (approximately 5 mg), weighed in quadruplicate, and placed in a stability chamber at 40 °C and 75% RH (relative humidity) for 3 months. The samples were collected at 2 weeks and 1, 2, and 3 months and prepared for analysis by adding an appropriate volume of selected media (water (CD complexes) or acetonitrile (PZQ)) to achieve a final concentration of 2 mg mL^–1^. Prior to LC-DAD analysis, the samples were filtered through a 0.2 μm PTFE filter mounted onto a syringe. At each time point, the *T*_0_ sample was prepared prior to analysis. For each degradant, the relative retention time was calculated (RRT = RT(degradation product)/RT(PZQ)).

### 2.9 Photostability Study

PZQ and its inclusion complexes with CDs were added into a glass screw cap vial (approximately 5 mg). Each sample was weighed in duplicate, and one of them was prepared as a control sample by wrapping the vial with aluminum foil. Both samples (test and control) were simultaneously exposed to the same stress conditions (300 W m^–2^, 1 h; 700 W m^–2^, 8 h). For each time point (*T*_0_ (prepared just before LC analyses) and *T*_1_ after 1 and 8 h), the samples were prepared by adding an appropriate volume of selected solvent (water for CD complexes or acetonitrile for PZQ) to achieve a concentration of 2 mg mL^–1^. For LC-DAD analysis, the samples were filtered through a 0.2 μm PTFE filter mounted onto a syringe.

### 2.10 HRMS Analysis of Degradation Products from Stability Studies

Samples from accelerated solid stress testing were further diluted (with acetonitrile (PZQ) and water CD complexes) to obtain concentrations of 10 and 100 μg mL^–1^, respectively.

## 3 Results and Discussion

### 3.1 Inclusion Complex Formation

#### 3.1.1 Phase Solubility Studies

Solubility studies of PZQ with selected CDs were investigated in water using liquid chromatography, UV–vis absorption spectroscopy, and fluorescence spectroscopy. The total concentration of PZQ was determined using validated methods (Table S1). Based on Higuchi and Connors,^14,15^ all observed phase diagrams were classified as A_L_ type (Figures S1–S3). The stability constants determined for 1:1 complexes correlate fairly well, although some discrepancies between UV–vis and other methods were observed. Based on these results, fluorescence spectroscopy can be suggested as the optimal method due to its low cost, high sensitivity, and overall ease of use.

The stability constants for β-CD, HPβCD, RMβCD, and SBEβCD and the corresponding complexation efficiencies are given in Table 1. The results suggest that RMβCD and SBEβCD have the most significant effect on the solubility of PZQ, with an increase of up to 16-fold observed in the presence of these CDs. Although the stability constant values obtained indicate efficient interaction between PZQ and the CDs, the binding is not too strong to limit dissociation from the complex and interfere with the drug absorption process after oral administration of the complex.^21^

**Table 1:** Stability Constants (*K*_1:1_), Complexation Efficiencies (CE), and Solubility Enhancement (*S*_max_/*S*_0_) for Binary Systems of PZQ with Selected CDs Obtained by Different Analytical Methods

		β-CD	HPβCD	RMβCD	SBEβCD
*K*_1:1_/M^–1^	HPLC	456.77 ± 19.68	282.18 ± 5.90	543.02 ± 1.70	487.55 ± 34.61
	UV–vis	422.63 ± 87.49	230.08 ± 43.05	483.64 ± 67.88	378.70 ± 47.40
	fluorescence	441.67 ± 15.86	281.30 ± 16.60	517.24 ± 45.07	517.68 ± 55.06
CE	HPLC	0.33 ± 0.03	0.21 ± 0.01	0.37 ± 0.01	0.34 ± 0.01
	UV–vis	0.33 ± 0.05	0.21 ± 0.01	0.34 ± 0.04	0.33 ± 0.03
	fluorescence	0.33 ± 0.03	0.23 ± 0.01	0.39 ± 0.01	0.35 ± 0.03
*S*_max_/*S*_0_	HPLC	5.26	10.63	16.58	15.44
	UV–vis	4.73	8.44	15.17	12.19
	fluorescence	5.09	9.51	15.79	16.37

The results obtained herein differ to some extent in comparison to previously reported data, which showed a significant level of discrepancy. For example, the stability constant for the binary system with β-CD determined by Becket et al.^9^ (396.91 M^–1^) differs from the one determined by Mourão et al.^13^ (275.56 M^–1^). Moreover, our results are not in agreement with research done by Münster et al.,^6^ where the stability constant for the binary system with SBEβCD of 365.11 M^–1^ was reported. These discrepancies in stability constant values could be explained by differences in analytical techniques used, as well as differences in experimental conditions, such as temperature, used during the study. However, the results reported in the same study^6^ for the binary system with HPβCD (228.71 M^–1^) are in relatively good agreement with our data.

Mass spectrometry, an efficient technique to study noncovalent complexes, can be used to determine the stoichiometry of host/guest complexes in the gas phase, which can be correlated with the behavior in solutions.^22,23^ Solutions of PZQ with selected CDs were analyzed by HRMS. Signals corresponding to 1:1 PZQ/CD complexes were observed indicating the formation of inclusion complexes (Table 2).

**Table 2:** Calculated and Measured *m*/*z* Values of Doubly Charged Adducts of PZQ/CD Complexes with Sodium Ions

	[M + 2Na]^2+^		
M	calculated	measured	mass error/ppm
PZQ + β-CD	746.266	746.2673	1.74
PZQ + HPβCD (DS 7)^a^	949.4125	949.4137	1.26
PZQ + RMβCD (DS 12)	830.3599	830.3602	0.36
PZQ + SBEβCD (DS 4)	1062.2687	1062.2672	–1.41

a DS, degree of substitution for CD derivative.

To confirm the actual inclusion complex formation and to reveal the drug–CD binding mode, the complexes were investigated in more detail by NMR spectroscopy. We have focused our NMR research on β-CD since the side chains of β-CD derivatives make the interpretation of NMR spectra more complicated.^24^

#### 3.1.2 Mechanism of Inclusion

PZQ and β-CD complex formation has been long established;^9,11,25−28^ however, a comprehensive and detailed analysis of a such system is still missing. Due to limited PZQ water solubility, our initial NMR experiments were performed in DMSO-*d*_6_, enabling the preparation of highly concentrated samples, and consequently fast acquisition and well-defined NMR spectra. However, the percentage of complexation in this solvent proved to be too low for any conclusions to be made (Figures S4–S14 and Table S2), possibly due to the solvation of PZQ with molecules of DMSO making the inclusion of the drug into the β-CD cavity unfavorable. Despite the challenges of low PZQ solubility, switching the study to D_2_O enabled investigation of drug–CD interactions in a medium resembling the biological system.

##### 3.1.2.1 Binding Information through Chemical Shift Perturbations

As a first step, we fully assigned proton and carbon chemical shifts of PZQ, β-CD, and PZQ/β-CD complex (Tables S3, S4, and 4), aiming to seek information about binding mode through chemical shift perturbations.

**Figure 1:**
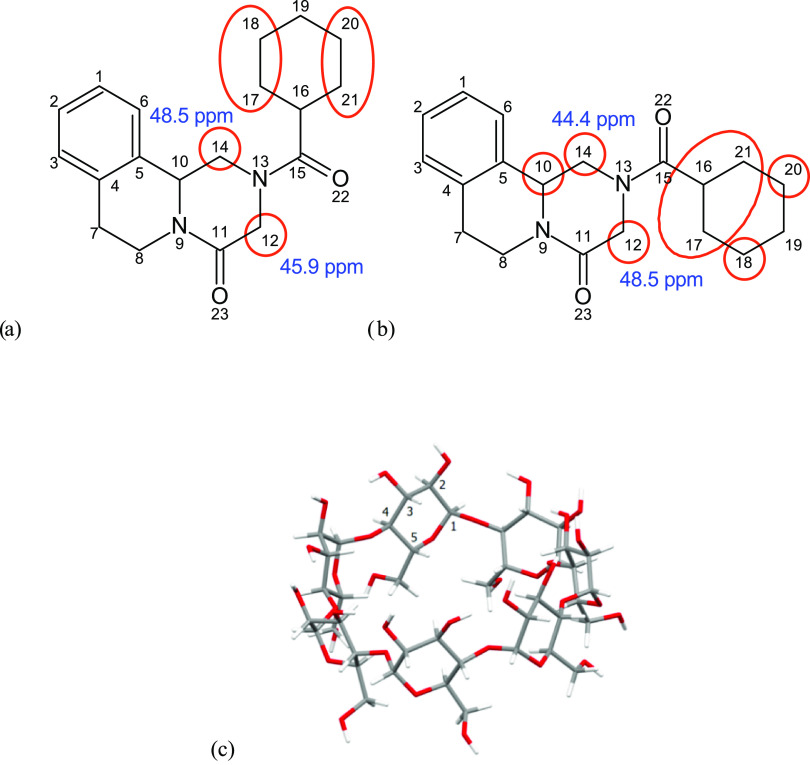
Two rotational isomers of PZQ: (a) *syn* and (b) *anti* key carbon chemical shifts marked in blue; areas of the biggest difference in chemical shifts between free and bound PZQ marked with red circles; (c) X-ray structure of β-CD with numbered atoms of one sugar unit.

As expected, the significant difference in chemical shifts (ca. 0.03 ppm) of β-CD (Table S3, Figures S15, and S16) was noticed only for protons H3 (free 3.892 ppm → complex 3.861 ppm) and H5 (free 3.787 ppm → complex 3.760 ppm), positioned on the inner side of the cyclodextrin central cavity (Figure 1c). This suggested PZQ insertion to the β-CD cavity and an inclusion complex formation.

Comparing the chemical shifts of free and bound PZQ (Tables 3 and S4) proved to be more challenging, not only due to low solubility but also, as previously seen in DMSO-*d*_6_ (full analysis can be found in the Supporting Information), as a result of PZQ undergoing a chemical exchange between two isomers (Figure 1a,b). Both were identified using their carbon chemical shifts (Table S4). The carbon chemical shift of C12 in *syn* rotamer was 45.9 ppm (Figure 1a), which is lower than the chemical shift of the same atom in *anti* isomer (48.5 ppm, Figure 1b) suggesting that the amide oxygen is in the vicinity of C12 in *syn*.^29^ Similarly, the chemical shift of C14 in the *anti* isomer (44.4 ppm, Figure 1b) was lower than its counterpart in *syn* (48.5 ppm, Figure 1a), corresponding to the oxygen atom being oriented toward C-14 in *anti*. The rotamer ratio was estimated to be 1:1 in D_2_O at 25 °C.

**Table 3:**
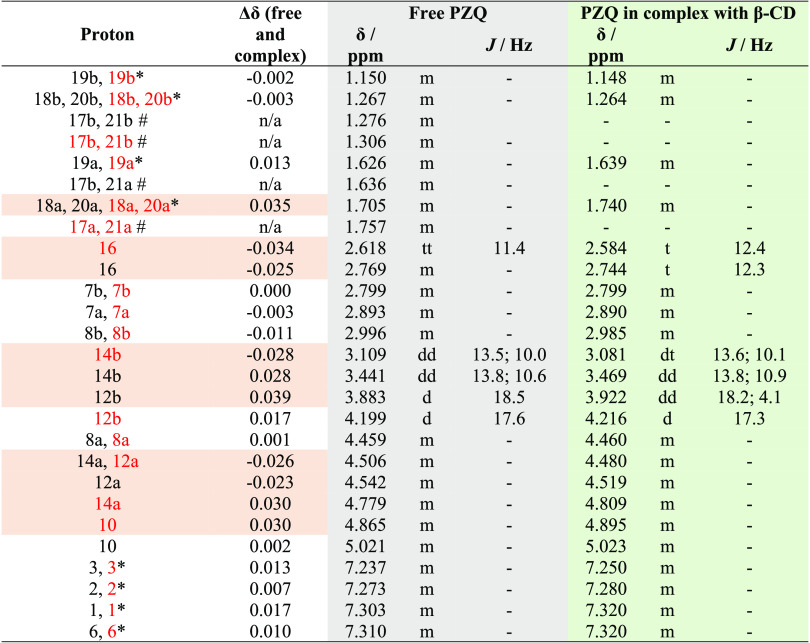
Comparison of Proton Chemical Shifts for Free PZQ and in Complex with β-CD; Atoms with the Largest Detected Difference Marked with Red Shading^a^

a *Due to the low solubility of PZQ and overlap, the proton chemical shifts for H1, H2, H3, H6, H18, H19, and H20 were extracted from HSQCe spectra; #H17 and H21 when in complex with β-CD exhibit large broadening of peaks in HSQCe, so chemical shifts could not be extracted at all.

**Figure 2:**
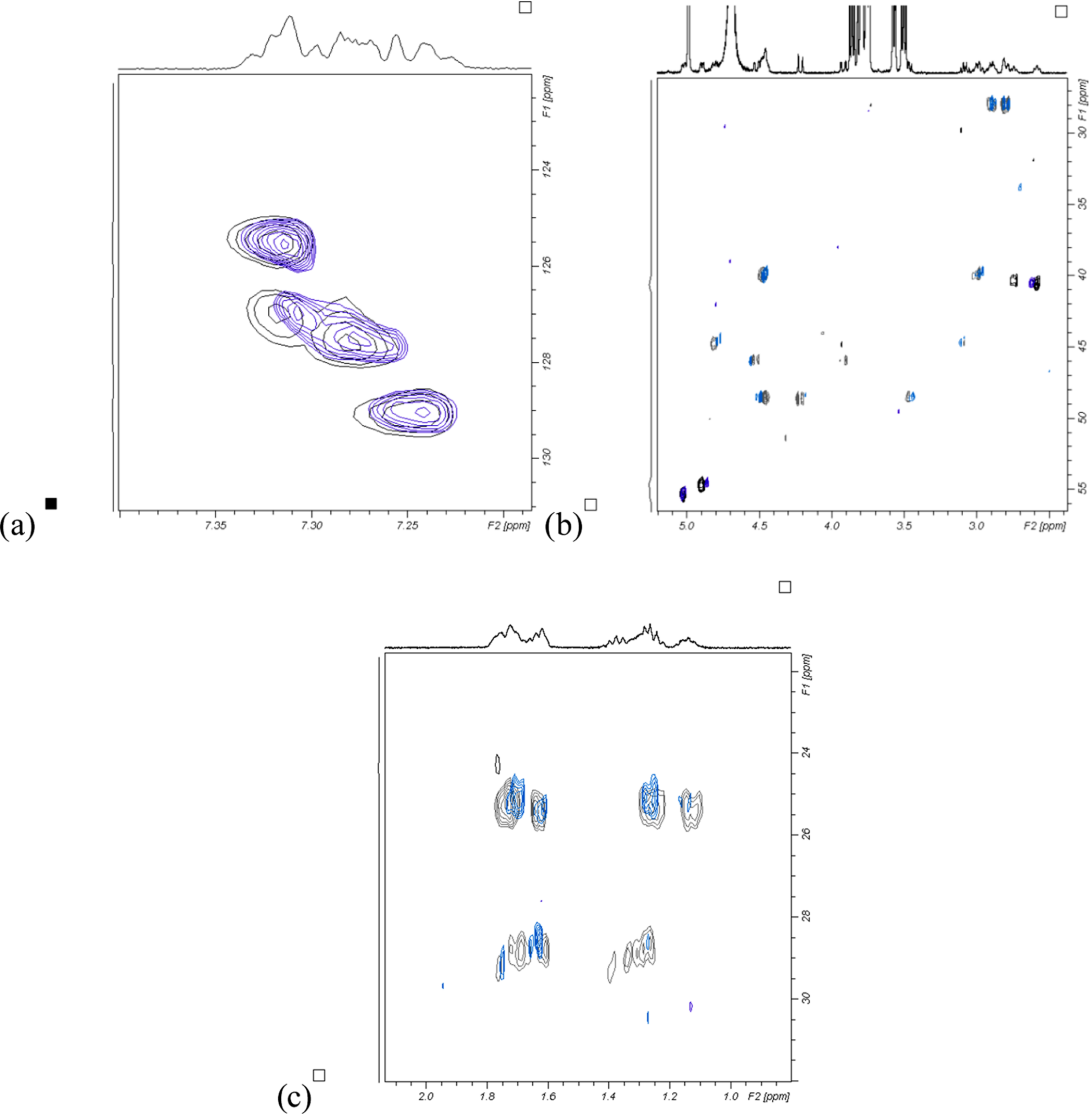
Comparison of the ^1^H–^13^C HSQCe spectra for PZQ (blue) and complex (black) in D_2_O at 25 °C: (a) aromatic region, (b) central region, and (c) cyclohexane ring region.

To circumvent all of these challenges, chemical shifts of PZQ were extracted using a combination of proton and ^1^H–^13^C HSQCe spectra (Figure 2). Although the spectral resolution of 0.007 ppm in the F2 domain of ^1^H–^13^C HSQCe spectrum was sufficient to extract proton chemical shifts, the spectral resolution in the F1 domain was ca 0.2 ppm, similar to the value of the observed effects, and therefore, ^13^C data could only be used as guidance (Table S4).

The atoms whose proton chemical shifts were most affected by the formation of inclusion complex with β-CD belong to the cyclohexane and central pyrazino ring. Additionally, when in complex, PZQ protons H17 and H21 could not be extracted, either from the proton spectrum due to the overlap or from the ^1^H–^13^C HSQCe spectrum due to line broadening (Figure 2c), again pointing to changes occurring in this part of the molecule. All of these findings suggest that cyclohexane and central pyrazino ring of PZQ might be in close contact with β-CD cavity.

#### 3.1.3 Intramolecular Interactions

The PZQ chemical shift perturbations observed are in agreement with differences reported by Rodrigues et al.^30^ during PZQ/methyl-β-cyclodextrin study.

**Figure 3:**
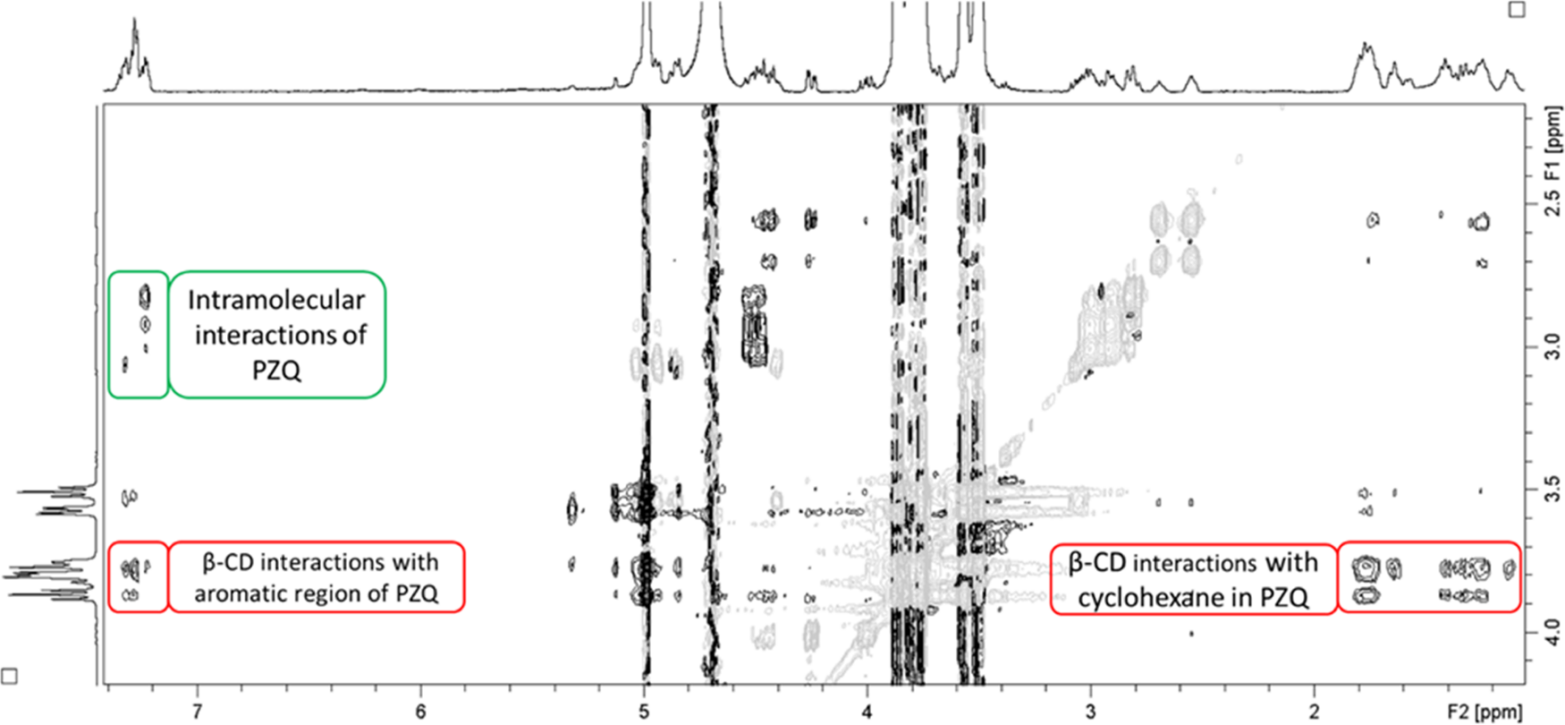
Region of the ROESY NMR spectrum of PZQ/β-CD complex in D_2_O at 25 °C showing interactions between the β-CD and PZQ (*c*(PZQ) = 3.52 mM and *c*(β-CD) = 14.08 mM).

However, our conclusions from chemical shift comparisons are not in accordance with PZQ orientation suggested earlier by de Jesus et al.^25^ When proposing the orientation of the PZQ aromatic part embedded within the β-CD cavity, the authors were guided by intermolecular interactions found in the ROESY spectrum (H3 and H5 of β-CD with aromatic part of the PZQ). Unfortunately, we were unable to observe these interactions with our PZQ/β-CD 1:1 complex both in the water suppression ROESY and ROESY spectrum recorded using the same experimental parameters as de Jesus et al. (Figures S18 and S19). As we strongly suspected that the main cause of signal absence was the low concentration, and in line with results obtained by phase solubility studies (Figures S1–S3), we prepared the solid mixture of PZQ/β-CD in a 1:4 molar ratio to achieve a much higher PZQ concentration in D_2_O. At a PZQ concentration of 3.52 mM, roughly 3 times higher than in complex at *c*(β-CD) = 1.07 mM, the resulting ROESY spectrum (Figure 3) showed not only expected interactions between the aromatic region of PZQ and β-CD but also a whole set of new, previously not reported, interactions between the cyclohexane part of PZQ and β-CD. This result was in perfect correlation with the chemical shift differences we observed in proton spectra.

##### 3.1.3.1 Measuring the Complexation Percentage and Association Constants with Diffusion Spectroscopy (DOSY)

**Figure 4:**
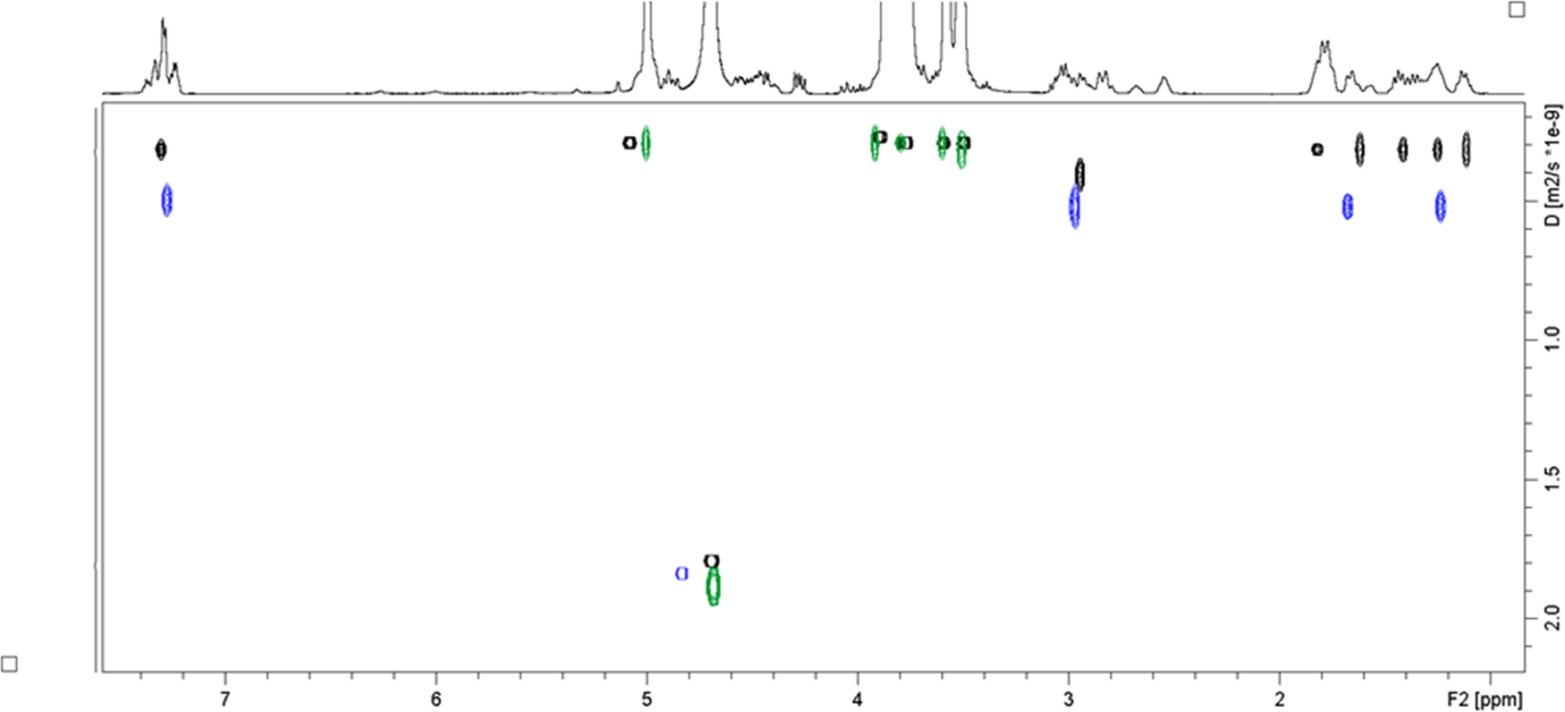
Overlay of the DOSY spectra: PZQ (blue), β-CD (green), and PZQ/β-CD complex at *c*(β-CD) = 14.08 M (black).

Encouraged by ROESY results and the obvious increase in PZQ solubility, we compared the complexation percentages at different β-CD concentrations. DOSY (Figure 4) was employed to measure the diffusion coefficients (D) for all species. The final diffusion coefficients were calculated as an average of all selected signals (Table 4).

**Table 4:** Diffusion Coefficients (*D*), Percentage of Complexation (*p*), and Association Constants (*K*_s_) Obtained from DOSY Experiments in D_2_O at 25 °C^a^^,^^b^

		*D* [m^2^/s]/10^–10^	error/10^–10^	p/%	*K*_s_/M^–1^
	PZQ—free	4.64	0.11		
	β-CD—free	2.57	0.04		
A	PZQ—complex	4.01	0.06	30.4	587
	β-CD—complex	2.54	0.01		
B	PZQ—complex	2.77	0.13	90.3	854
	β-CD—complex	2.41	0.02		

a A—at 1.07 mM β-CD.

b B—at 14.08 mM β-CD.

The results in Table 4 suggest that the diffusion coefficient of β-CD is not perturbed by interaction with PZQ. Lower diffusion coefficients of PZQ in the complexes can be explained by some percent of bound PZQ in the mixture undergoing fast exchange (on DOSY scale) with free PZQ. When these two conditions are satisfied, it is possible to calculate the percentage of complexation (*p*) using the equation^31−33^
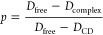
The percentages of complexed PZQ with β-CD in D_2_O were calculated to be ca. 30% (at 1.07 mM β-CD) and ca. 90% (at 14.08 mM β-CD). A literature search revealed only one previously reported DOSY experiment,^11^ performed on a 1:1 PZQ/β-CD complex reporting 37% of complexation.

Subsequently, the percentage of complexed PZQ (*p*) can be used to determine association constant (*K*_s_) in the following equation^31^
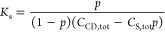
where *C*_CD,tot_ and *C*_S,tot_ are total concentrations of β-CD and PZQ, respectively. Association constants were calculated to be 587 and 854 M^–1^, at 1.07 and 14.08 mM β-CD, respectively. The former value is in good agreement with the association constant obtained in the phase solubility study (Table 1), especially considering large errors in diffusion coefficients.

##### 3.1.3.2 Molecular Modeling

**Figure 5:**
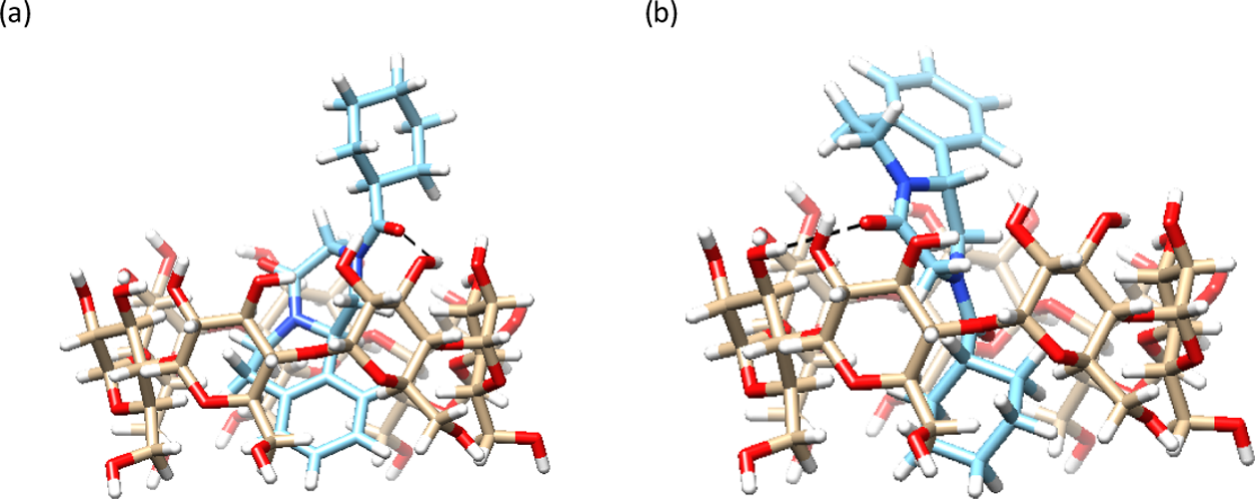
Docking study binding modes in which β-CD cavity is occupied with: (a) aromatic part or (b) cyclohexane and central pyrazino ring of PZQ. Hydrogen bonds are labeled as dashed lines.

Aiming to explain the discrepancy between the previously postulated mode of inclusion described in the literature^25^ and the one obtained from our NMR results, molecular modeling was performed independently of any experimental NMR data. The docking study resulted in 15 binding modes, which can be grouped into (i) ones with the PZQ aromatic part embedded within the β-cyclodextrin cavity (Figure 5a) and (ii) ones with the cyclohexane and central pyrazino ring of PZQ within the β-CD cavity (Figure 5b). Relative binding energies calculated by docking (Table S5) are not sufficiently different to enable identification of the dominant binding mode. Besides van der Waals interactions, hydrogen bonds between β-CD and carbonyl group of central pyrazino ring of PZQ can also be formed.

These molecular modeling results completely explained our NMR experimental results:

(a) Diffusion spectroscopy (DOSY) detects the difference in molecules according to their diffusion rates. Although free PZQ and the complex exhibit vastly different diffusion rates, complexes with different binding modes, all simultaneously present in solution, display an average diffusion rate due to the fast exchange between the different binding modes. Similarly, in proton and carbon chemical shifts of β-CD, the effect of perturbation is cumulative and it only testifies about the drug occupying the cavity.
(b) On the other hand, the ROESY spectrum would show a different set of intermolecular interactions for each mode of binding. Considering the low solubility of PZQ at 1.07 mM β-CD, the presence of two different isomers in exchange, and several different modes of binding, the concentration of each type of complex becomes so small that it falls below the detection limit of the NMR method, thereby explaining the absence of intramolecular interactions in ROESY spectra under such conditions. If the solution concentration and percentage of complexation are increased (by higher β-CD concentration), the threshold is overcome and the interactions become visible.
(c) Finally, in addition to the previously reported aromatic part of PZQ,^25^ our NMR results showed that PZQ cyclohexane and central pyrazino rings are also in close contact with β-CD. This corresponds perfectly to several different binding modes all simultaneously present in solution, as identified by molecular modeling.

Since the CD cavity size is recognized as the critical parameter determining the drug–CD binding mode,^34^ one can assume a similar structure of other complexes of PZQ with CDs examined.

### 3.2 Effect of CDs on PZQ Chemical Stability

Our previous study^12^ suggested that CD complexation of PZQ may compromise its chemical stability. Therefore, in the second part of the study, the effect of CDs on PZQ stability at different conditions was examined, and the structures of formed degradation products were proposed.

#### 3.2.1 Stress Conditions

Samples collected during degradation stress conditions (HCl, NaOH, and H_2_O_2_), analyzed via UPLC-DAD-MS, revealed both known and novel degradation products, not yet described in the literature. Known degradation products (*m*/*z* 203 and 331 for HCl (Figure S20),^35^*m*/*z* 331 for NaOH, and *m*/*z* 329 and 345 for H_2_O_2_^36−38^ (Table S7)) were observed in PZQ and PZQ/CD complexes. Under oxidative stress conditions, some unknown degradation products were also observed: *m*/*z* 148 and 217, both for PZQ and PZQ/CD complexes, and *m*/*z* 215 for PZQ/CD complexes and are described in detail in the following section.

#### 3.2.2 Accelerated Solid Stress Conditions

**Figure 6:**
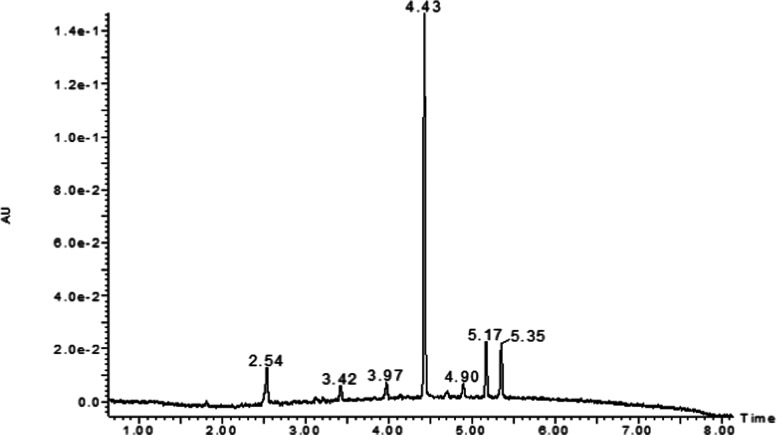
UPLC-DAD chromatogram of PZQ/HPβCD and degradation products from accelerated solid stress (3 months at 40 °C and 75% RH).

Following storage of PZQ (*m*/*z* 313) and PZQ/CD complexes under accelerated solid stress conditions for up to 3 months at 40 °C and 75% RH, several degradation products were detected (Figure 6). Relative amounts of degradation products, expressed as a percentage of peak area, are listed in Table 5. After 3 months, the highest level of degradation (38%) was observed for PZQ/HPβCD, while PZQ/RMβCD and PZQ/SBEβCD remained stable with only minor degradation (ca. 4 and 6%, respectively). The difference in the level of degradation can be related to the determined stability constants of the complexes formed (Table 1). The lower *K*_1:1_ value corresponds to the higher degradation level, in line with results reported for spironolactone degradation catalyzed by CDs.^24^ Assuming a similar PZQ binding mode with all CD derivatives examined, determined mostly by the dimensions of the CD central cavity, it appears that the presence of the functional groups on the CD core has a critical impact on the chemical stability and the degradation level of PZQ within the complexes formed. Jarho et al.^24^ demonstrated that the number of hydroxyl groups available for reaction is one of the key factors determining the relative catalytic activity of CDs. This is probably the main reason behind high drug degradation levels observed for the PZQ/HPβCD complex. On the contrary, the number of available hydroxyl groups in RMβCD and SBEβCD is reduced, leading to a higher chemical stability of PZQ in such complexes.

**Table 5:** Relative Amount of Detected Degradation Products of PZQ and PZQ/CD Complexes Formed Following Storage under Accelerated Solid Stress Conditions (40 °C and 75% RH for 3 Months)^a^

		% peak area									
analyte	time	RRT 0.57	RRT 0.60	RRT 0.77	RRT 0.90	RRT 0.95	RRT 1.10	RRT 1.14	RRT 1.17	RRT 1.23	PZQ
PZQ	*T*_0_					0.14			1.83	0.14	97.90
	2 weeks					0.14			2.32	0.26	97.28
	1 month								2.31	0.17	97.52
	2 months								2.28		97.72
	3 months								2.18		97.82
PZQ/HPβCD	*T*_0_				1.05				1.60	1.21	92.86
	2 weeks	1.72			1.10		1.18	1.14			93.51
	1 month	4.35			2.99		0.73	0.47	2.21	4.46	81.81
	2 months	6.29	0.58	2.07	3.12		1.13		5.37	5.10	74.25
	3 months	8.88		2.73	3.22		2.99		8.87	8.94	62.54
PZQ/RMβCD	*T*_0_								1.47		98.53
	2 weeks	0.30		0.35	0.16				1.51		97.68
	1 month	0.83							1.39	0.41	96.52
	2 months	1.11		0.94					0.97	1.38	94.49
	3 months	0.96		0.83					1.48	0.62	96.11
PZQ/SBEβCD	*T*_0_								1.44		96.97
	2 weeks	1.03	0.97	0.45	1.51				2.10		93.94
	1 month	2.21			1.22					1.35	94.07
	2 months	2.33							4.61		93.06
	3 months	0.83							5.03		94.14

a RRT = RT (degradation product)/RT(PZQ).

A similar set of degradation products was detected for all three PZQ/CD complexes. However, analogue peaks corresponding to some of them were completely absent in pure PZQ chromatograms, suggesting that CD complexation promotes new degradation pathways, as most pronounced in the PZQ/HPβCD complex. LC-HRMS and MS/MS analyses were used to annotate the molecular formula and to provide structural information of degradation products. Fragments obtained from MS/MS studies (Figure S21), as well as proposed structures for degradation products, are summarized in Table 6.

**Table 6:**
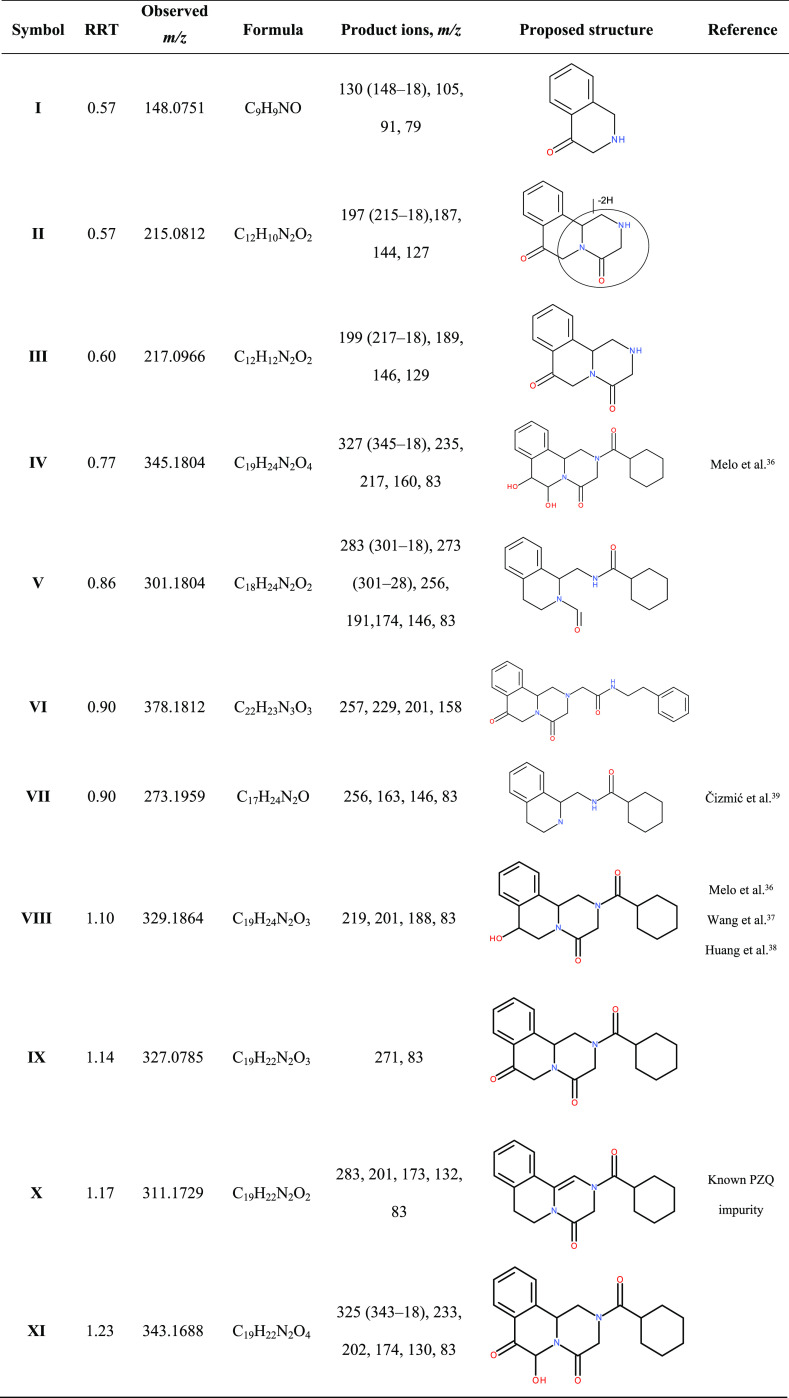
List of Detected Degradation Products for PZQ and PZQ/CD Complexes Formed Following Storage under Accelerated Solid Stress Conditions (40 °C and 75% RH for 3 Months)^a^

a RRT = RT (degradation product)/RT(PZQ).

Among the degradation products observed, several were previously reported in the literature with fragment ions at *m*/*z* 345 (IV), 273 (VII), 329 (VIII), and 311 (X), detected at relative retention times (RRT) 0.77, 0.90, 1.10, and 1.17, respectively.^36−39^ The degradation product at RRT 0.77 (*m*/*z* 345) corresponding to dihydroxypraziquantel was detected in all three PZQ/CD complexes. The MS spectrum displayed fragment *m*/*z* 327, while fragment *m*/*z* 309 was absent. The only dihydroxypraziquantel with such fragmentation pattern reported so far is 7,8-dihydroxypraziquantel.^36^ Due to the specific position of two hydroxy groups in the molecule, upon elimination of the first molecule of water (*m*/*z* 345 → 327), the resulting enol tautomerizes into a more stable keto form preventing the loss of the second water molecule. This was further confirmed by the presence of fragment *m*/*z* 217, which corresponds to the loss of one water molecule and a cyclohexylketene.

The degradation product detected at RRT 0.90 (*m*/*z* 273), potentially the result of amide bond hydrolysis, was also detected in all three complexes. Fragment ions observed in MS/MS spectra are in agreement with previously published results^39^ with *m*/*z* 256 formed due to oxygen loss and *m*/*z* 163 formed due to the loss of cyclohexylketene following the cleavage of the amide bond.

A monohydroxylated structure was suggested for degradation product RRT 1.10 (*m*/*z* 329), which was only detected for the PZQ/HPβCD complex, based on observed fragments and previously reported data.^36−38^ The fragment ion *m*/*z* 311 would correspond to the loss of water; however, it was not detected in MS/MS spectra possibly due to the high fragmentation of the parent ion. The presence of fragment ions *m*/*z* 219, 201, and 83 suggests oxidation in the hexahydropyrazinoisoquinoline part of PZQ, instead of the cyclohexyl ring. As previously reported,^36^ position 7, where C–H bonds are benzylic and thereby most likely a preferred point of oxidation, is the most reactive site in PZQ.

The degradation product at RRT 1.17 (*m*/*z* 311) was detected in all formulations and during most sample collection points up to 3 months. Previously, *m*/*z* 311 was proposed to be the product of dehydrogenation of the hexahydropyrazinoisoquinoline moiety with the exact position of the double bond undetermined.^38,40^ However, as fragment *m*/*z* 311 could also belong to a known impurity of PZQ, 1,2-deshydro praziquantel (MW 310, Figure S22a), this was later confirmed in retention time with UPLC-DAD via the commercially available standard.

In addition to known degradants, the exact mass and structure were proposed for seven new degradation products, based on HRMS, which have not been previously published in the literature, and are summarized in Table 6. These included degradants detected at RRT 0.57 (*m*/*z* 148 and *m*/*z* 215), RRT 0.60 (*m*/*z* 217), RRT 0.86 (*m*/*z* 301), RRT 0.90 (*m*/*z* 273 (previously published) and *m*/*z* 378), RRT 1.14 (*m*/*z* 327), and RRT 1.23 (*m*/*z* 343).

The degradant at RRT 0.57 was detected in all three PZQ/CD complexes, whereas the RRT 0.60 degradant was detected only in PZQ/HPβCD and PZQ/SBEβCD. Both degradation products give fragment ions arising from loss of one water molecule (*m*/*z* 197 and 199) and loss of CO from the parent ion (*m*/*z* 215 → 187 and *m*/*z* 217 → 189). All fragments in MS/MS spectra of degradation product *m*/*z* 215 suggest one additional double bond, compared with fragment ions from degradation product *m*/*z* 217. However, the data were insufficient to accurately define the position of the double bond. In addition, a second degradation product was observed at RRT 0.57 (*m*/*z* 148, accurate mass of [M + H]^+^ 148.0751), for which the proposed molecular formula is C_9_H_9_NO with fragment ions corresponding to the loss of one water molecule (148 → 130), likely at position 7 as the preferred point of oxidation.

The degradation product at RRT 0.86 (*m*/*z* 301, accurate mass of [M + H]^+^ 301.1804, proposed molecular formula of C_18_H_24_N_2_O_2_) was only detected using UHPLC-QTOF-MS due to lower sensitivity. MS/MS spectra were similar to degradation product *m*/*z* 273 (RRT 0.90). Fragmentation of the parent ion (*m*/*z* 301) showed an elimination of 28, assigned to CO, and formation of fragment ion *m*/z 273. Fragment ion *m*/*z* 256 is formed due to oxygen loss. Cleavage of the amide bond gives fragment ion *m*/*z* 191. In MS/MS spectra of degradation product *m*/*z* 273, cleaving amide bond gives fragment ion *m*/*z* 163. The difference between these two fragment ions corresponds to the molecular weight of a CO group. Melo et al. proposed a degradation mechanism of PZQ and formation of fragment ions *m*/*z* 174 and 146.^36^ Following cleavage of the bond on the opposite side of the nitrogen atom in amide bond, fragment ion *m*/*z* 174 is formed. Fragment ion *m*/*z* 146 corresponds to the loss of the CO group (*m*/*z* 174 → 146), suggesting that the position of a CO group could be at the nitrogen atom in the pyrazine ring.

The RRT 1.14 degradant was only detected in the PZQ/HPβCD complex, with an accurate mass of [M + H]^+^ 327.0785 and molecular formula C_19_H_22_N_2_O_3_. A parent ion with *m*/*z* 327 was previously reported,^36,37^ but results were not in agreement with our MS/MS spectra. Melo et al.^36^ proposed a ketone at position 19 on the cyclohexyl ring with fragment ions *m*/*z* 327 → 203 → 174 → 146. Furthermore, Wang et al.^37^ proposed two dehydrogenated monooxidized structures with fragment ions *m*/*z* 201, 144, and 130. In our case, the two major fragment ions (*m*/*z* 217 and 83) suggest a ketone on the hexahydropyrazinoisoquinoline part of PZQ. As previously described, position 7 is most likely to be the preferred point of oxidation.

Degradation product *m*/*z* 343 was detected at RRT 1.23 (accurate mass of [M + H]^+^ 343.1688, C_19_H_22_N_2_O_4_, Table 2) in all three PZQ/CD complexes. Fragment *m*/*z* 325 corresponds to the loss of one water molecule. Fragment ion *m*/*z* 83 suggests that there is no change on the cyclohexyl part of molecule. Fragment ion *m*/*z* 233 corresponds to the cleavage of the amide bond and loss of cylcohexylketene.

The two degradation products found at RRT 0.90 included previously discussed *m*/*z* 273 and *m*/*z* 378 (accurate mass of [M + H]^+^ 378.1812, C_22_H_23_N_3_O_3_). This large molecule could be explained by a known impurity of PZQ (MW 363, Figure S22b). Accurate mass and fragment ions in MS/MS spectra correspond to the oxidation of impurity and the formation of ketone. Fragment ion *m*/*z* 257 is assigned to the cleavage of the amide bond. Following cleavage of the bond on the opposite side of the CO group, fragment ion *m*/*z* 229 is formed. Fragment ion *m*/*z* 201 is assigned to the cleavage between nitrogen from the piperazine ring and carbon atom and loss of one water molecule. A small amount of impurity MW 363 was found in neat PZQ (*m*/*z* 343, RRT 0.95) (Table 5), providing confirmation that the detected degradation product could arise from a PZQ impurity and not PZQ itself.

#### 3.2.3 Photostability Study

All PZQ complexes tested appear stable under photostability conditions at 300 W m^–2^ for 1 h, as well as under conditions at 700 W m^–2^ for 8 h, except for PZQ/SBEβCD with a decay of about 35% (Table 7). All detected degradation products are previously found during the accelerated solid stress testing.

**Table 7:** Relative Amount of Detected Degradation Products of PZQ/SBEβCD Formed under Photostability Study Conditions (700 W m^–2^ for 8 h)

		% peak area					
analyte	photostability conditions	RRT 0.58	RRT 0.60	RRT 0.78	RRT 0.90	RRT 0.93	PZQ
PZQ/SBEβCD	700 W m^–2^	5.59	1.81	5.00	2.34	4.72	75.12

All of the structures of degradation products in this manuscript are proposed on the basis of experimental data obtained through HRMS and fragmentation patterns from MS/MS. To unambiguously determine the structures of herein identified degradation products, our next step includes a more rigorous larger-scale forced degradation study of PZQ complexes, which would enable isolation of degradation products and confirmation by subsequent NMR analysis.

## 4 Conclusions

In this work, we combined several methodologies to characterize PZQ/CD complexes, with respect to solubility and chemical stability, as well to investigate and elucidate mechanisms of complex formation. Mechanisms of complex formation were investigated and confirmed via NMR and modeling, and novel degradants reported following accelerated solid stress studies.

Phase solubility studies showed a significant increase in the solubility of PZQ in the presence of β-cyclodextrin and its hydroxypropyl, randomly methylated, and sulfobutylether derivatives. The formation of 1:1 inclusion complexes was confirmed by HRMS.

NMR studies have confirmed that under given conditions (D_2_O, 25 °C), complexes of PZQ and β-CD can be detected, with a calculated percentage of complexation of ca. 30 and 90% at β-CD concentrations of 1.07 and 14.08 mM, respectively. Chemical shift perturbations and analysis of ROESY NMR spectra confirmed that the cyclohexane and central pyrazino ring, as well as the previously suggested^25^ aromatic part of PZQ, are in close contact with β-CD through several different binding modes. These findings were confirmed by molecular modeling.

Accelerated stress testing of solid PZQ/CD complexes for up to 3 months displayed a decrease in PZQ stability in the presence of CDs. PZQ/HPβCD showed the highest level of degradation after 3 months (38%), while PZQ/RMβCD and PZQ/SBEβCD remained relatively stable with only minor degradation. A similar set of degradation products was detected across all three PZQ/CD complexes, some of which were previously reported in the literature. In addition, seven new degradation products were detected and the corresponding structures were proposed based on HRMS and MS/MS data, indicating that CD complexation promoted new degradation pathways of the drug.

Data from accelerated solid stress testing should be considered when storing PZQ cyclodextrin formulations, avoiding high temperatures and high humidity.

## Supporting Information Available

Phase solubility data; one dimensional (1D) and two dimensional (2D) NMR spectra; molecular modeling; and MS/MS spectra of degradation products (PDF)
