# Supplementary material for: Investigation of Praziquantel/Cyclodextrin Inclusion Complexation by NMR and LC-HRMS/MS: Mechanism, Solubility, Chemical Stability, and Degradation Products
Source: Mol Pharm. 2021 Oct 21;18(11):4210–23. doi: 10.1021/acs.molpharmaceut.1c00716 (PMC8564759; doi:10.1021/acs.molpharmaceut.1c00716)
Supplement: Supplementary file 1 — mp1c00716_si_001.pdf [file mp1c00716_si_001.pdf]

Investigation of praziquantel/cyclodextrin inclusion complexation by NMR and LC-HRMS/MS: mechanism, solubility, chemical stability and degradation products

Tatjana Kezele Špehar, Marijana Pocrnić, David Klarić, Branimir Bertoša, Ana Čikoš, Mario Jug, Jasna Padovan, Snježana Dragojević, and Nives Galić

Supporting Information

Contents

1. Phase solubility 2
  1.1. Validation of methods 2
  1.2. Phase solubility diagrams 2
2. Full assignment of β-cyclodextrin in DMSO-d_6 4
3. Full assignment of praziquantel in DMSO-d_6 4
4. Praziquantel and β-cyclodextrin complex in DMSO-d_6 6
  4.1. Chemical shift comparisons 6
  4.2. Analysis of the NOESY spectrum 8
  4.3. Analysis of the 1D NOESY and 1D ROESY spectra 8
  4.4. Diffusion Spectroscopy (DOSY) 9
5. Praziquantel and β-cyclodextrin complex in D_2O 11
  5.1. Spectra for chemical shift analysis 11
  5.2. ROESY spectra 14
6. Molecular modeling 16
7. Linearity of UPLC-DAD method used for identification of the degradation products 16
8. Stress conditions 17
9. Accelerated solid stress testing 18

1. Phase solubility

1.1. Validation of methods

Table S1. Validation of methods for quantification of praziquantel (PZQ)

	HPLC	UV-Vis	Fluorescence
linearity	y = 90.254x + 19.288	y = 1.149x + 0.004	y = 33.664x + 58.568
	R^2 = 0.9996	R^2 = 0.9999	R^2 = 0.9991
range / μg mL^{-1}	2 – 100	10 – 1600	0 – 25
LOD / μg mL^{-1}	0.06	8.22	0.40
LOQ / μg mL^{-1}	0.17	24.91	1.22

1.2. Phase solubility diagrams

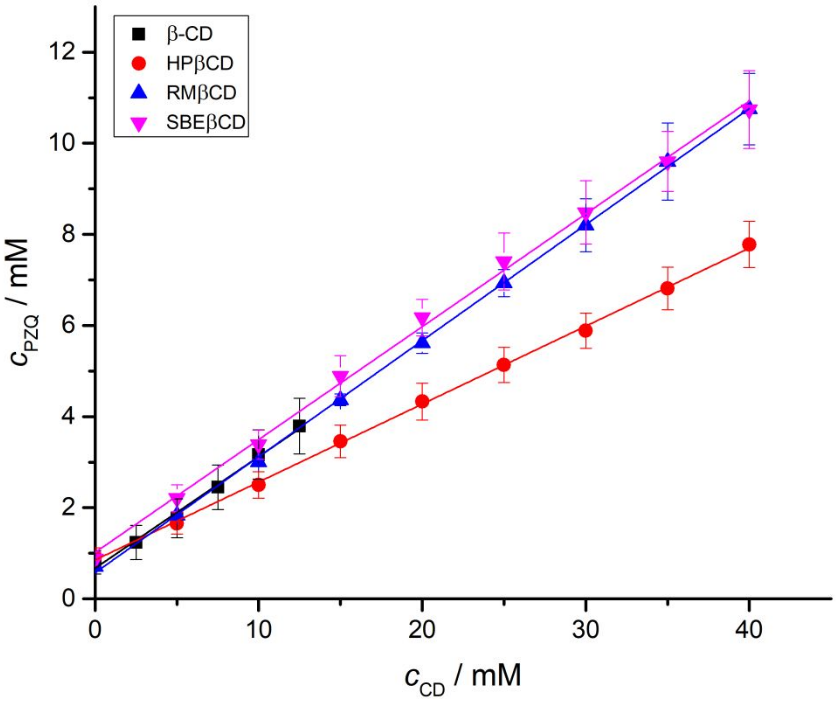

Figure S1. Phase solubility diagrams of PZQ with β-CD(■), HPβCD(●), RMβCD(▲) and SBEβCD(▼) in water at ambient temperature, UV-VIS as method of quantification, solvent methanol:water 1:4.

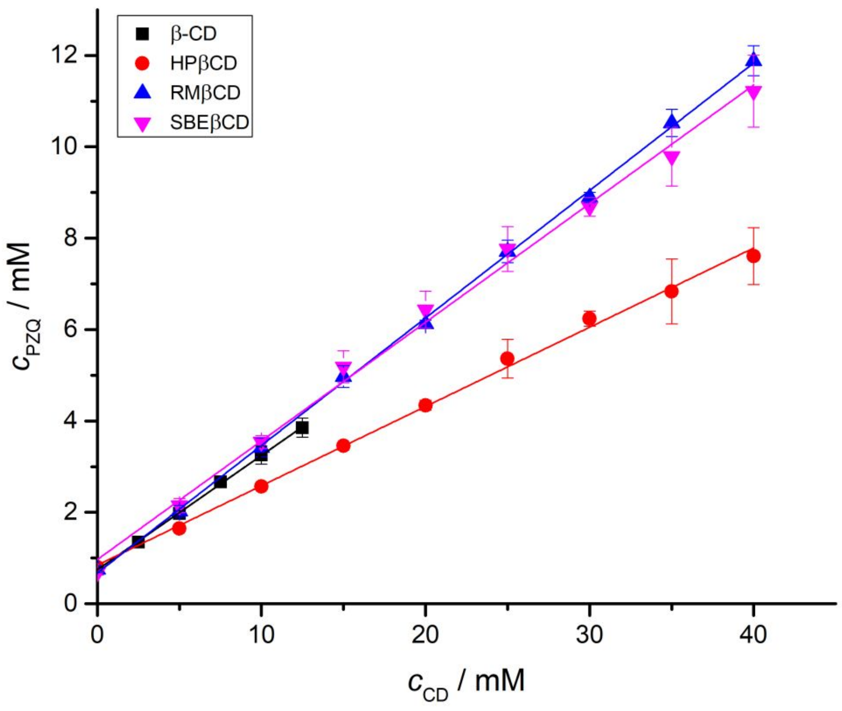

Figure S2. Phase solubility diagrams of PZQ with β-CD(■), HPβCD(●), RMβCD(▲) and SBEβCD(▼) in water at ambient temperature, fluorescence spectroscopy as method of quantification, solvent methanol:water 1:4.

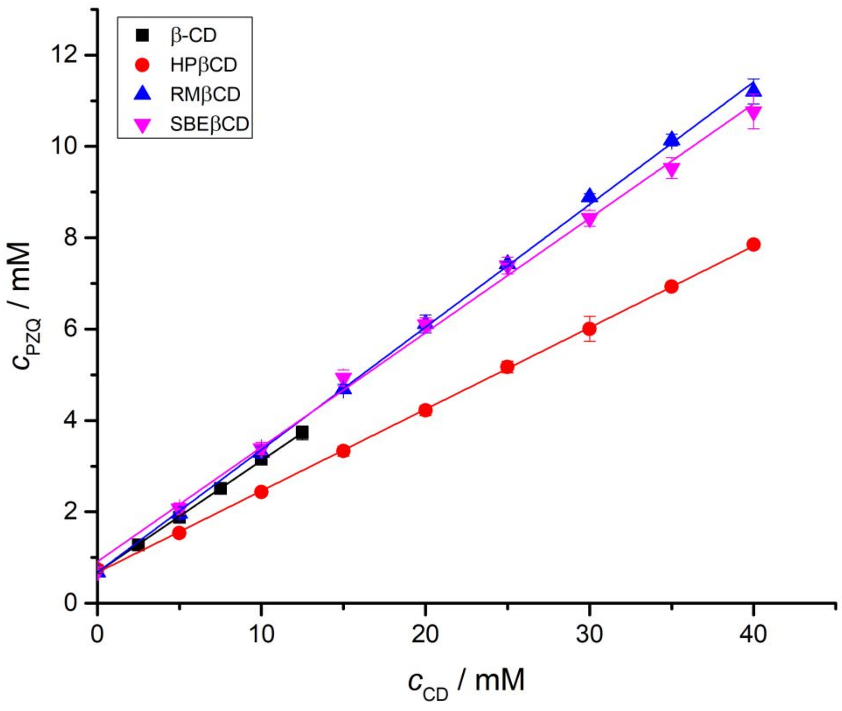

Figure S3. Phase solubility diagrams of PZQ with β-CD(■), HPβCD(●), RMβCD(▲) and SBEβCD(▼) in water at ambient temperature, HPLC as method of quantification, mobile phase as solvent.

2. Full assignment of β-cyclodextrin in DMSO-d_6

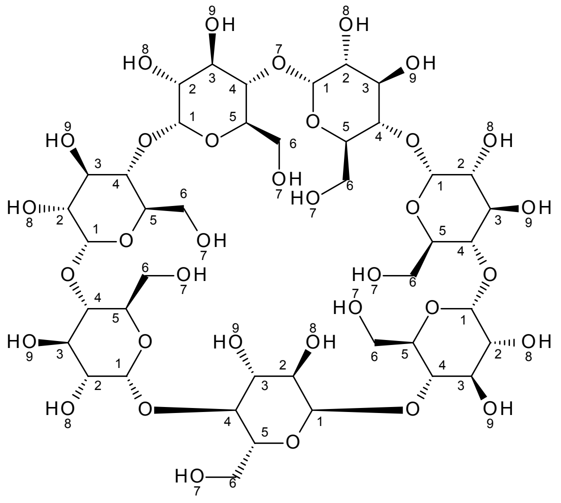

Figure S4. Structure and numbering for β-CD.

^{1}H NMR (600 MHz, DMSO-d_6): δ = 3.30 (ddd, J = 9.7, 6.4, 3.7 Hz, 7 H, 2), 3.35 (t, J = 9.4 Hz, 7 H, 4), 3.56 (dt, J = 11.0, 3.0, 2.0 Hz, 7 H, 5), 3.58 - 3.64 (m, 7 H, 6b), 3.62 - 3.65 (m, 7 H, 3), 3.64 - 3.69 (m, 7 H, 6a), 4.45 (t, J = 5.7 Hz, 7 H, 7), 4.83 (d, J = 3.7 Hz, 7 H, 1), 5.67 (d, J = 2.6 Hz, 7 H, 9), 5.72 (d, J = 7.0 Hz, 7 H, 8) ppm.

^{13}C NMR (151 MHz, DMSO-d6): δ = 59.9 (6), 72.0 (5), 72.4 (2), 73.0 (3), 81.5 (4), 101.9 (1) ppm.

3. Full assignment of praziquantel in DMSO-d_6

NMR analysis of 1D and 2D NMR spectra recorded in DMSO-d_6 at 25 °C revealed two sets of resonance lines which both correspond to the proposed structure (Figure S5), their ratio being 55% : 45%.

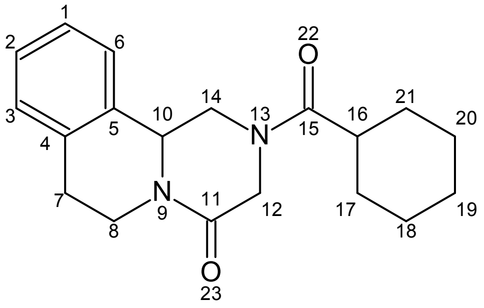

Figure S5. Structure and numbering for PZQ.

Variable temperature experiments showed coalescence of most signals at 80 °C (Figure S6), showing that two sets of resonance lines belong to two conformational isomers in chemical exchange. This conclusion was further corroborated by exchange peaks between the two conformers observed in NOESY spectrum (Figure S7).

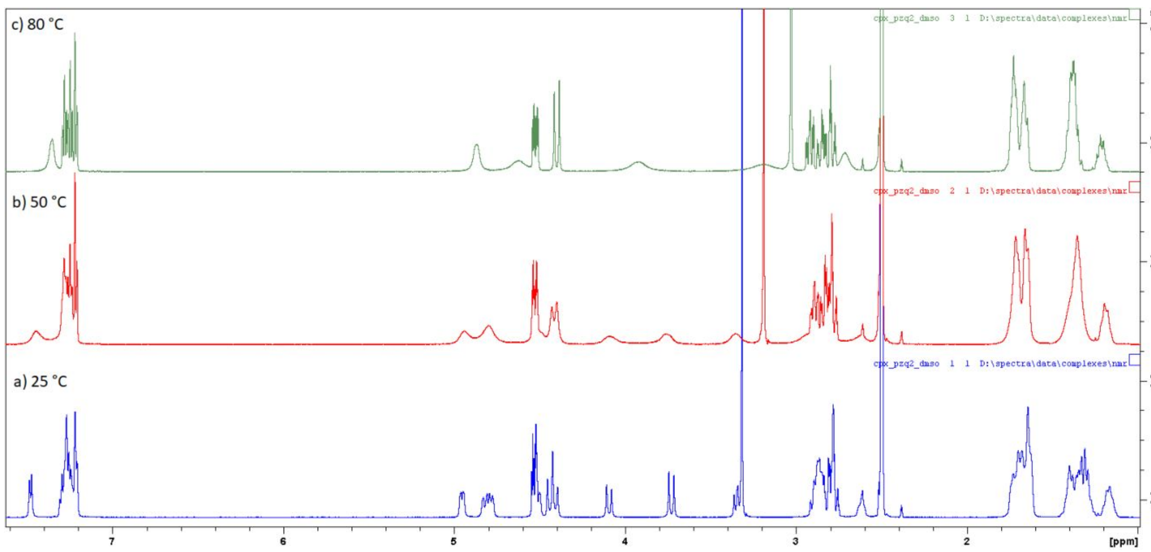

Figure S6. VT proton NMR spectra of IJ-11 showing coalescence of signals with temperature increase.

When comparing the two isomers, it immediately comes to view that the biggest difference in chemical shifts is around N13-C15=O amide bond, pointing towards these conformational isomers being the consequence of slow rotation around the amide bond.

Chemical shift for C12 in major rotamer (ca 55%) is 45.6 ppm, which is lower than the chemical shift of the same atom (48.4 ppm) in minor isomer (ca 45%). This suggests that amide oxygen is in vicinity of C12 in case of major rotamer.[1]

Similarly, chemical shift of C14 (44.4 ppm) in minor rotamer is lower than its counterpart in major rotamer (48.0 ppm). This corresponds oxygen atom being oriented towards C-14 in minor isomer.

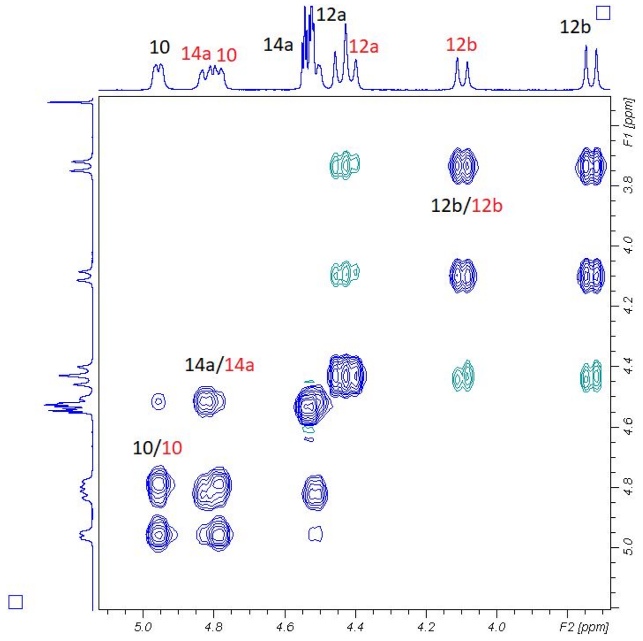

Figure S7. Part of NOESY spectrum for PZQ in DMSO-d_6 at 25 °C showing exchange peaks (blue) between the two conformers.

^{1}H NMR (600 MHz, DMSO-d$_6$): δ = 1.17 (br. s., 2 H, 19b), 1.23 - 1.47 (m, 8 H, 20b, 18b, 21b, 17b), 1.59 - 1.79 (m, 10 H, 19a, 20a, 18a, 21a, 17a), 2.61 (dt, 1 H, 16b), 2.74 - 2.82 (m, 4 H, 7<">, 7<'>), 2.82 - 2.95 (m, 4 H, 8<">, 16b, 14<">), 3.34 (dd, *J* = 13.3, 11.1 Hz, 1 H, 14<">), 3.73 (d, *J* = 17.8 Hz, 1 H, 12<">), 4.10 (d, *J* = 17.3 Hz, 1 H, 12<">), 4.41 (br. s., *J* = 18.0 Hz, 1 H, 12<'>), 4.44 (d, *J* = 17.8 Hz, 1 H, 12<'>), 4.51 (br. s., *J* = 13.3, 3.0 Hz, 1 H, 14<'>), 4.53 (dd, *J* = 11.7, 3.7 Hz, 2 H, 8<'>), 4.79 (dd, *J* = 10.2, 3.0 Hz, 1 H, 10), 4.82 (dd, *J* = 13.3, 3.0 Hz, 1 H, 14<'>), 4.96 (dd, *J* = 10.2, 3.1 Hz, 1 H, 10), 7.19 - 7.23 (m, 2 H, 3), 7.23 - 7.32 (m, 5 H, 6, 1, 2), 7.48 (d, *J* = 7.6 Hz, 1 H, 6) ppm.

^{13}C NMR (151 MHz, DMSO-d$_6$): δ = 24.9 (20), 24.9 (18), 25.5 (19), 28.1 (7), 28.7 (17), 28.9 (21), 29.0 (21), 29.2 (17), 38.2 (8), 38.3 (8), 38.7 (16), 39.1 (16), 44.4 (14), 45.6 (12), 48.0 (14), 48.4 (12), 53.9 (10), 54.8 (10), 125.3 (6), 125.9 (6), 126.5 (1), 126.6 (1), 127.0 (2), 127.1 (2), 129.0 (3), 133.2 (5), 133.5 (5), 134.9 (4), 135.1 (4), 164.1 (11), 164.7 (11), 173.7 (15), 173.9 (15) ppm.

4. Praziquantel and β-cyclodextrin complex in DMSO-d$_6$

4.1. Chemical shift comparisons

Comparison of proton and carbon chemical shifts was performed using the DMSO-d$_6$ solutions of PZQ, β-CD and praziquantel/β-CD mixture obtained as solid from the mill, showing no difference in resonance lines in either proton, or carbon spectra. The comparison is shown in Figures S8, S9, S10 and S11, the result suggests that the complex was not formed.

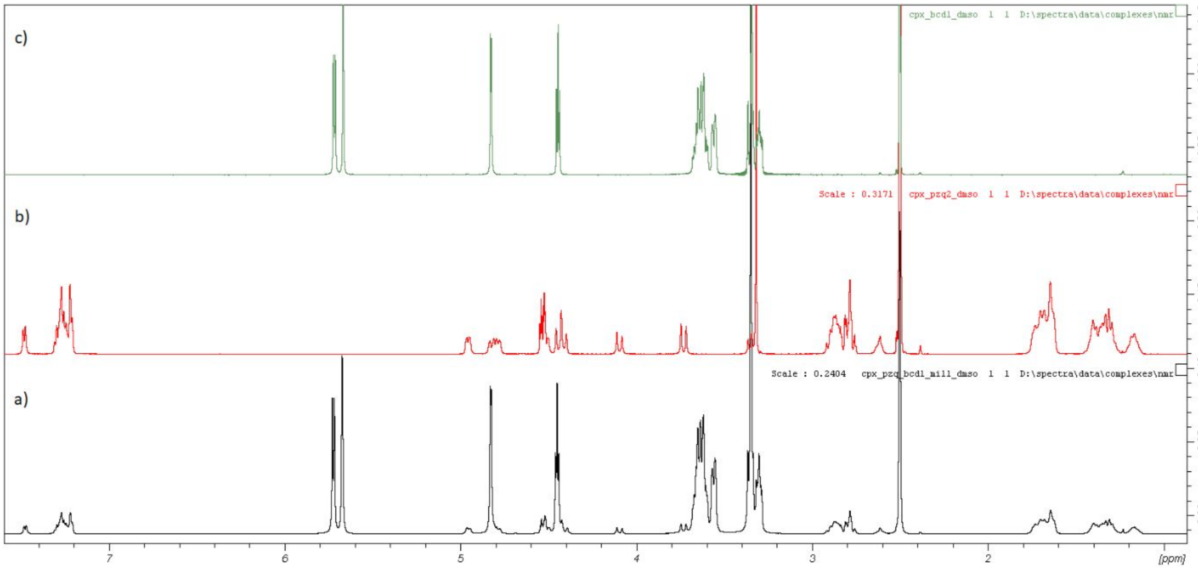

Figure S8. Comparison of the ^{1}H spectra for a) PZQ/β-CD solid mixture, b) PZQ and c) β-CD in DMSO-d$_6$ at 25 °C.

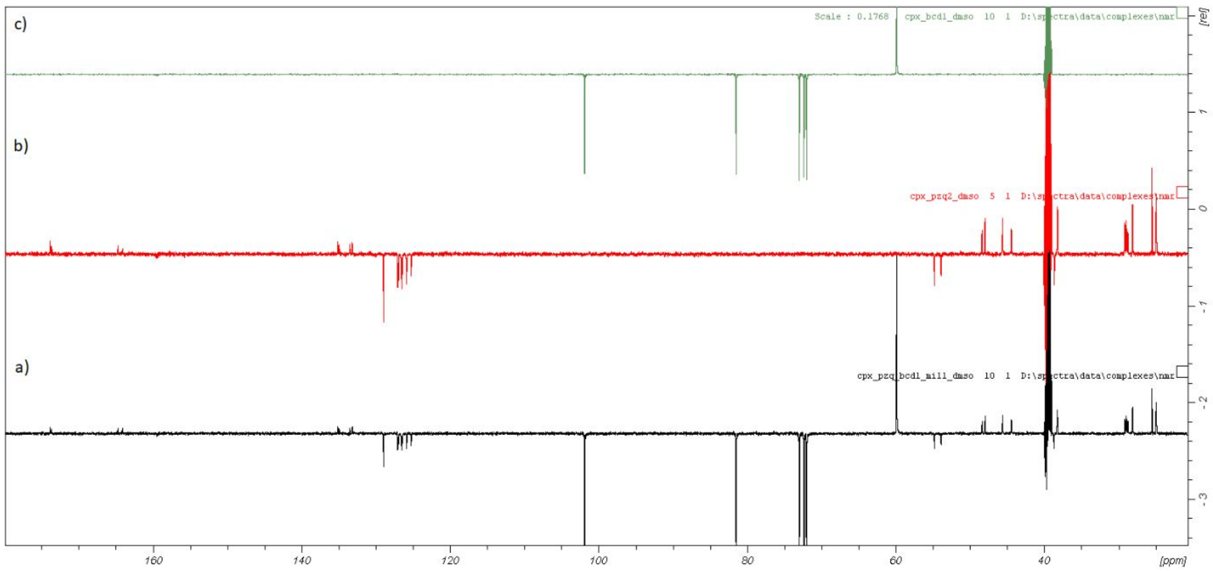

Figure S9. Comparison of the ^{13}C spectra for a) PZQ/β-CD solid mixture, b) PZQ and c) β-CD in DMSO-d_6 at 25 °C.

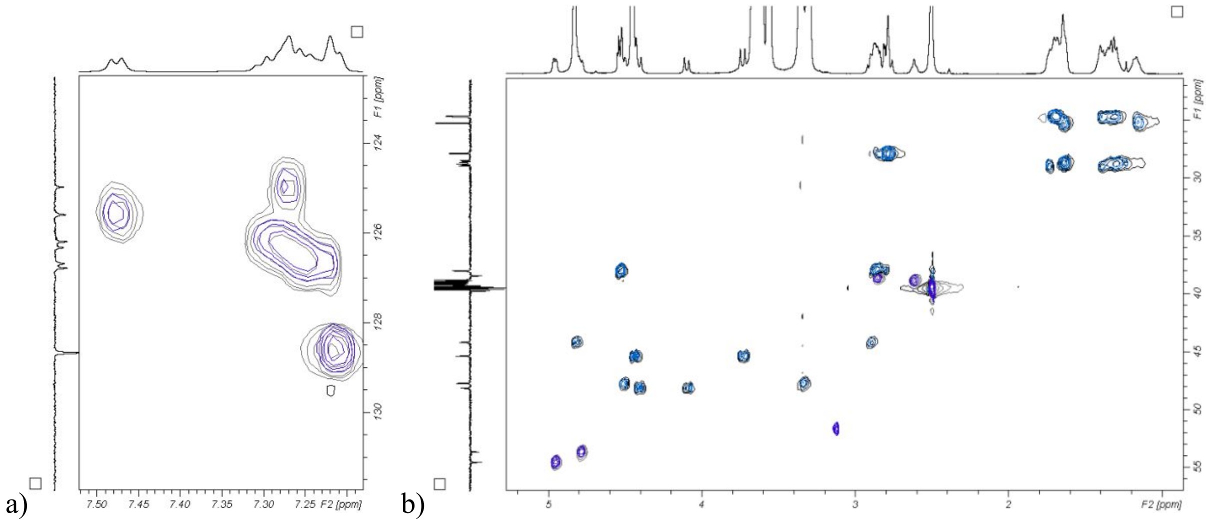

Figure S10. HSQCe spectra overlay of PZQ (blue) and the PZQ/β-CD solid mixture (black): a) aromatic, b) aliphatic region; DMSO-d_6 at 25 °C.

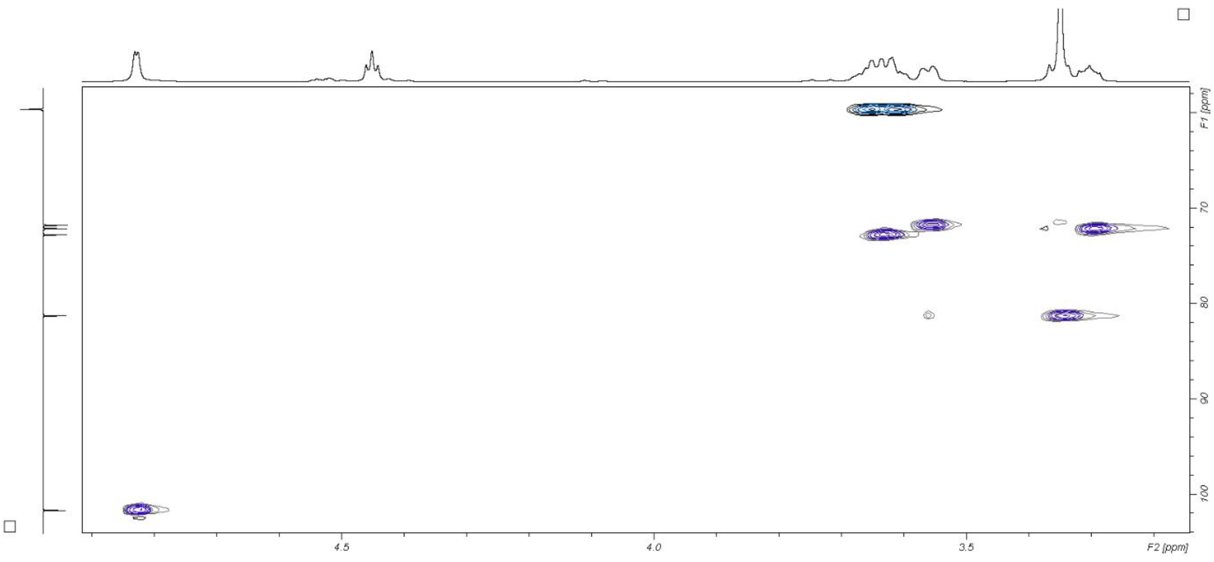

Figure S11. HSQCe spectra overlay of β-CD (blue) and the PZQ/β-CD solid mixture (black) in DMSO-d_6 at 25 °C.

4.2. Analysis of the NOESY spectrum

NOESY spectrum of the solid mixture showed two sets of signals, one being negative (Figure S12, green) and the other positive (Figure S12, black). Negative signals belong to β-CD and positive signals to PZQ. The two species show no mutual interactions, indicating that no complex was formed.

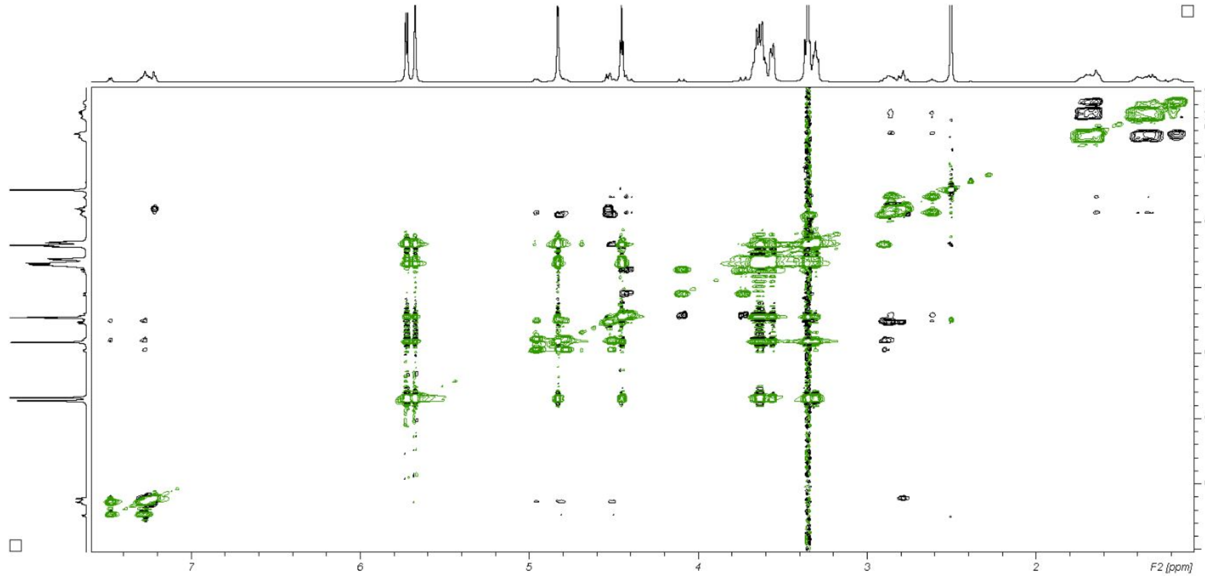

Figure S12. NOESY spectrum of PZQ (black) and β-CD (green) solid mixture in DMSO-d_6 at 25 °C.

4.3. Analysis of the 1D NOESY and 1D ROESY spectra

Figure S13 shows an overlay of the proton spectrum with selectively excited 1D NOESY and 1D ROESY spectra. Resonances selected for excitation were 3.63 ppm for H3 (Figure S13, b and c) and 3.56 ppm for H5 (Figure S13, d and e) of the β-CD. Selective ROESY spectra showed artefacts of TOCSY transfer within the β-CD, but neither of the performed experiments showed any interactions between β-CD and the drug molecule.

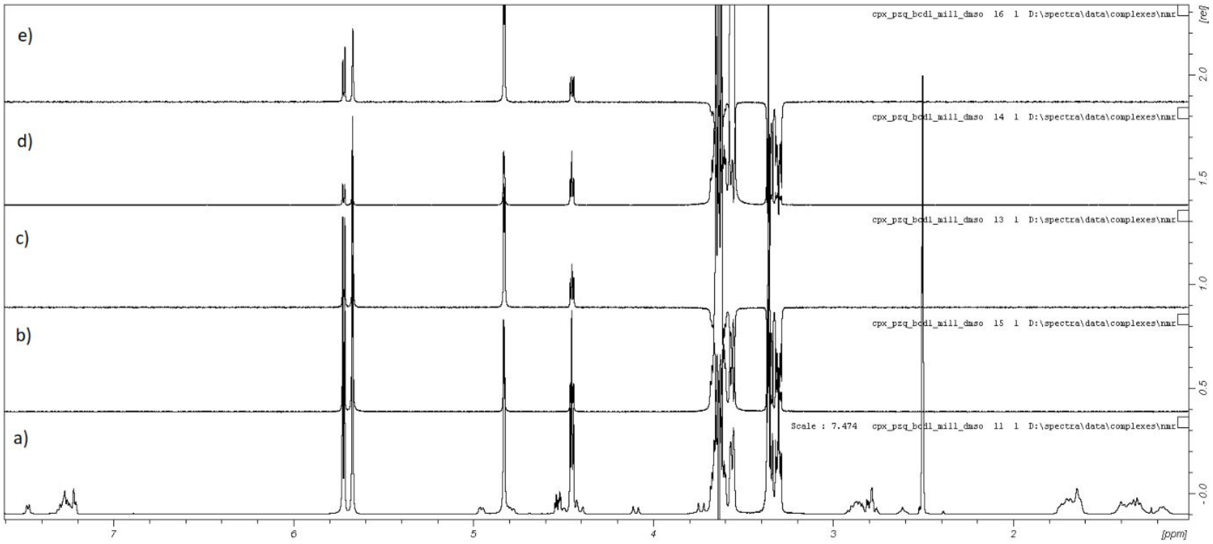

Figure S13. Overlay of a) ^{1}H, b) 1D NOESY with selective excitation at 3.63 ppm (H3,H6-βCD), c) 1D ROESY with selective excitation at 3.63 ppm (H3,H6-βCD), d) 1D NOESY with selective excitation at 3.56 ppm (H5-βCD), e) 1D ROESY with selective excitation at 3.56 ppm (H5-βCD) in DMSO-d_6 at 25 °C.

4.4. Diffusion Spectroscopy (DOSY)

Diffusion spectroscopy was used to measure the diffusion coefficients (D) of all species in the mixture, as well as the percentage of complexation. After the baseline correction, signals were integrated and automatically processed. The fitted curves were examined and only the signals with good fit were taken for further calculations. The omitted signals were overlapped and/or signals with low signal : noise ratio resulting in large integration errors. The final diffusion coefficient was calculated as average of all selected signals. The results are summarised in Table S2 and graphical depiction is shown in Figure S14.

Table S2. Diffusion coefficients (D) obtained from DOSY experiments in DMSO-d_6 at 25 °C

	D [m^2/s] / 10^{-10}	Error / 10^{-10}
PZQ – free	2.55	0.03
PZQ – bound	2.23	0.03
β-CD – free	0.99	0.01
β-CD – bound	1.00	0.04

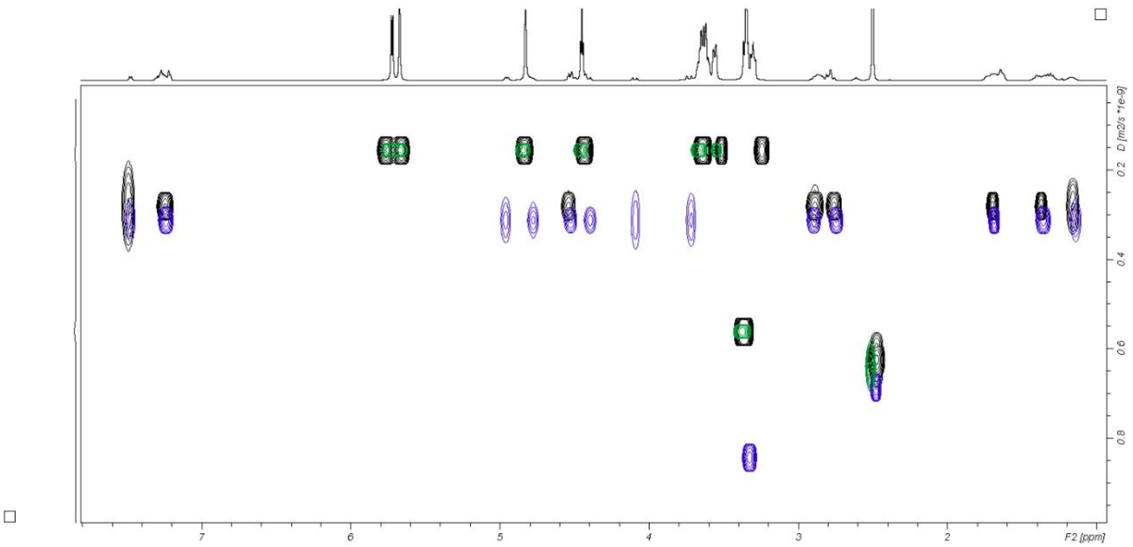

Figure S14. Overlap of the DOSY spectra: PZQ (blue), β-CD (green) and PZQ and β-CD solid mixture (black).

From Table S2. it is visible that the diffusion coefficient of β-CD is not perturbed by possible interaction with PZQ. Slightly lower diffusion coefficient of PZQ in the mixture can be explained by some percent of bound PZQ in the mixture undergoing the fast exchange (on DOSY scale) with the free PZQ. When these two conditions are satisfied, it is possible [2–4] to calculate percentage of complexation from following equation:

$$f_{complexed} = \frac{D_{free} - D_{complex}}{D_{free} - D_{CD}}$$

Substituting values from Table S2, the percentage of complexed PZQ with β-CD in DMSO-d_6 was calculated to be 20%. This low percentage can explain the lack of intermolecular interactions in NOESY/ROESY spectra, as well as no noticeable chemical shifts differences in proton and carbon spectra.

5. Praziquantel and β-cyclodextrin complex in D_2O

5.1. Spectra for chemical shift analysis

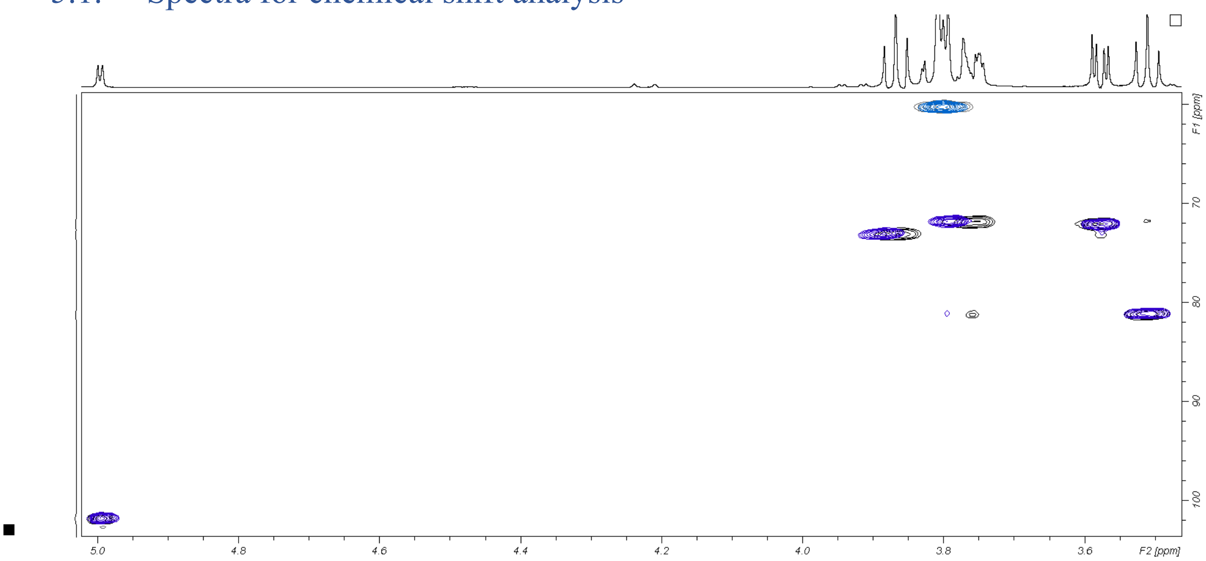

Figure S15. Comparison of the ^1H-^{13}C HSQCe spectra for β-CD (blue) and complex (black) in D_2O at 25 °C.

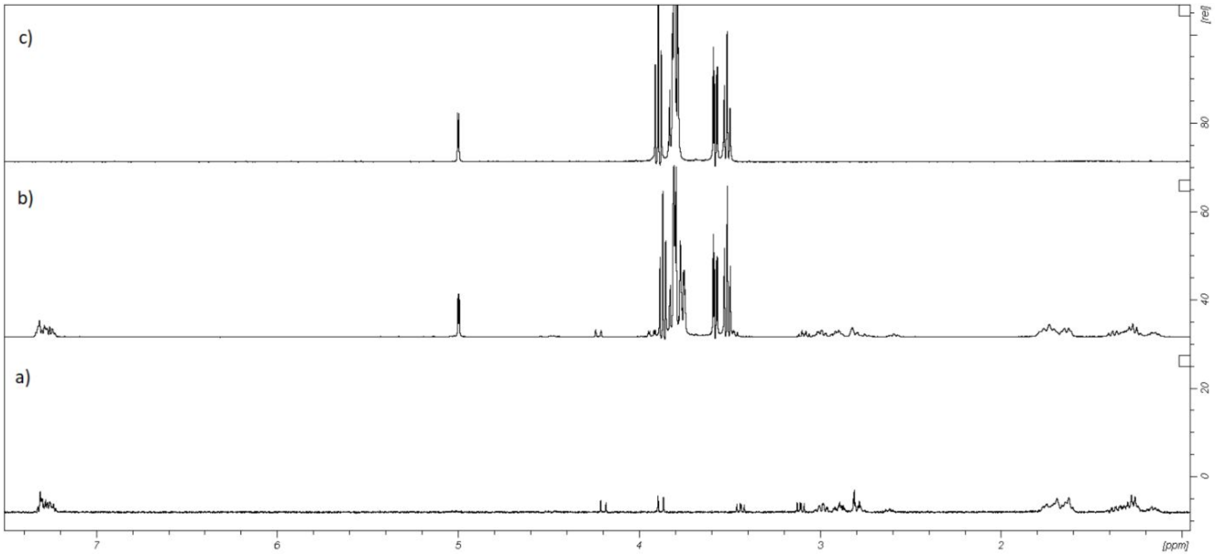

Figure S16. Comparison of the ^1H spectra for a) PZQ, b) PZQ and β-CD, c) β-CD in D_2O at 25 °C.

Table S3. Comparison of proton and carbon* chemical shifts for free β-CD and in complex with PZQ, atoms with biggest detected difference marked with red shading

**Proton**	**Δδ (free and complex)**	**Free βCD**			**βCD in complex with PZQ**			**Carbon**	**Δδ (free and complex)**	**Free βCD***	**βCD in complex with PZQ***
		**δ / ppm**		***J* / Hz**	**δ / ppm**		***J* / Hz**			**δ / ppm**	**δ / ppm**
4	-0.008	3.511	t	9.4	3.503	t	9.5	6	-0.1	60.3	60.2
2	-0.006	3.577	dd	10.3; 3.8	3.571	dd	10.3. 3.8	5	0.0	71.8	71.8
5	-0.026	3.787	m	-	3.760	m	-	2	-0.1	72.1	72.0
6	0.012	3.789	m	-	3.801	m	-	3	0.1	73.1	73.2
3	-0.031	3.892	t	9.7	3.861	t	9.7	4	0.0	81.1	81.1
1	-0.008	4.997	d	3.8	4.989	d	3.8	1	0.0	101.9	101.9

* carbon chemical shifts were extracted form HSQC spectrum with resolution of ca 0.2 ppm

Table S4. Comparison of carbon chemical shifts extracted from HSQCe spectra for free PZQ and in complex with β-CD, atoms with biggest detected difference marked with red shading; #H17 and H21 when in complex with β-CD exhibit large broadening of peaks in HSQCe, so chemical shifts could not be extracted at all

Carbon	Δδ (free and complex)	Free PZQ	PZQ in complex with βCD
		δ / ppm	δ / ppm
20, 18, 20, 18	0.2	25.1	25.3
19, 19	0.1	25.4	25.4
7, 7	0.1	27.8	28.0
17, 21#	n/a	28.7	-
21, 17 #	n/a	29.1	-
8, 8	0.0	39.9	39.9
16	-0.1	40.4	40.3
16	0.0	40.5	40.5
14	0.3	44.4	44.7
12	0.0	45.9	45.9
12, 14	0.1	48.5	48.5
10	0.1	54.5	54.6
10	-0.1	55.4	55.3
6, 6	0.0	125.5	125.5
1, 1	0.0	127.0	127.0
2, 2	0.1	127.5	127.6
3, 3	0.1	129.0	129.1
Red = *anti* isomer; black = *syn* isomer			

5.2. ROESY spectra

Analysis of PZQ ROESY spectrum in D_2O at 25 °C (Figure S17) yielded no additional information on the conformation of the free compound. All observed nOe contacts were expected and belong to neighbouring protons. Comparison to the ROESY spectrum of the complex revealed couple of additional nOe interactions, which are probably the result of slight change in chemical shifts resulting in less overlap or less accidental water suppression after the binding.

Analysis of PZQ/β-CD complex ROESY spectrum (Figure S18) unfortunately did not reveal any intermolecular interactions which would confirm the formation of the complex or the orientation of the PZQ within the β-CD cavity.

NOESY spectrum was also recorded, but showed no usable data, most probably due to the complex undergoing zero-crossing of the signals under experimental conditions.

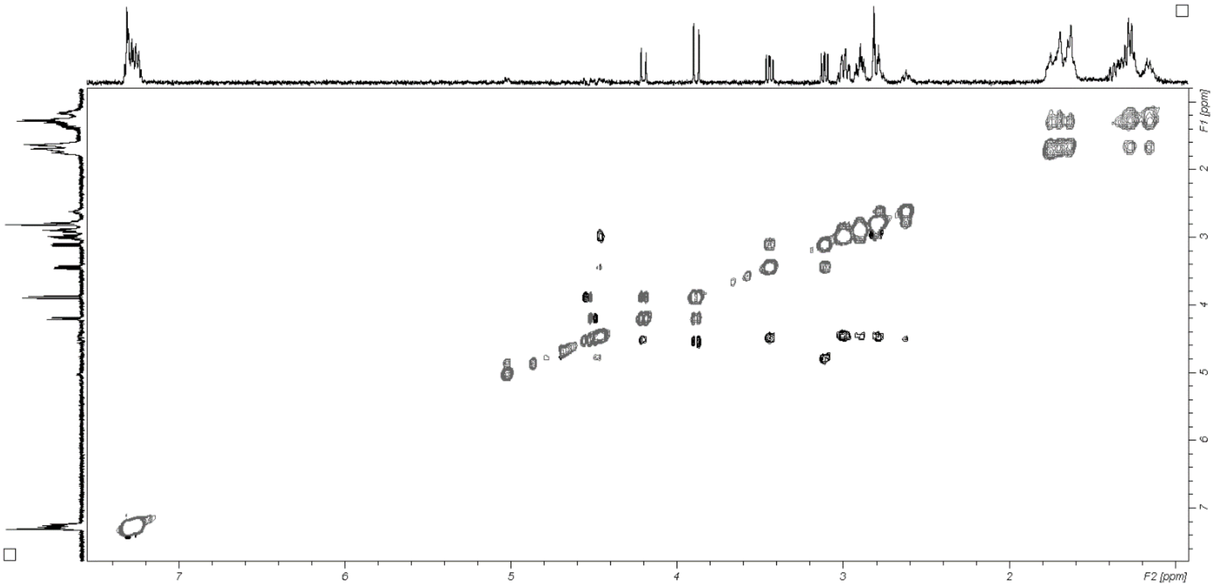

Figure S17. ROESY spectrum of PZQ in D_2O at 25 °C, water suppression using 3-9-19 pulse sequence.

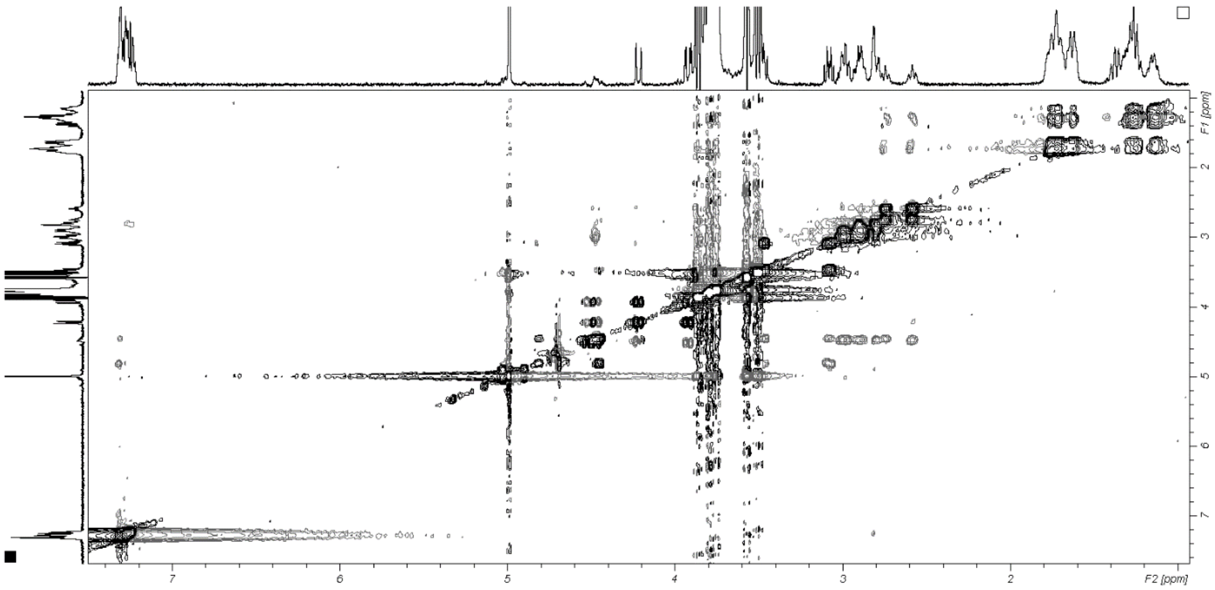

Figure S18. ROESY spectrum of PZQ and β-CD complex in D_2O at 25 °C, water suppression using 3-9-19 pulse sequence.

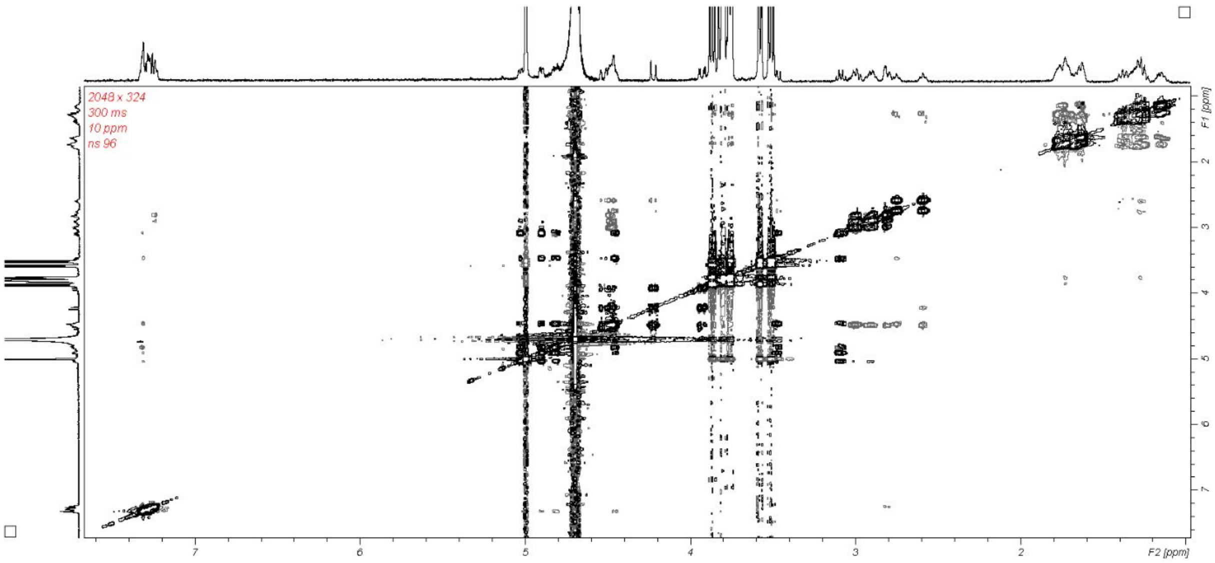

Figure S19. ROESY spectrum of PZQ and β-CD mixture in D_2O at 25 °C, without water suppression.

6. Molecular modeling

Table S5a. Relative binding energies of PZQ (R stereoisomer) calculated by docking study

mode[1]	affinity (kcal/mol)
A	-6.2
A	-6.1
B	-6.0
C	-5.9
A	-5.8
A	-5.8
B	-5.8
A	-5.8
B	-5.8
A	-5.7
C	-5.6
C	-5.5
A	-5.5
A	-5.4
C	-5.4

[1] modes: A - the ones with PZQ aromatic part imbedded within the β-cyclodextrin cavity (Figure 5a in the manuscript), B - the ones with cyclohexane and central pyrazino ring of PZQ within the β-CD cavity (Figure 5b in the manuscript), C - in-between modes A and B.

Table S5b. Relative binding energies of S – stereoisomer of PZQ calculated by docking study.

mode[1]	affinity (kcal/mol)
C	-6.2
B	-6.1
A	-6.1
B	-6.0
A	-6.0
B	-6.0
B	-5.9
A	-5.8
A	-5.8
A	-5.7
A	-5.5
A	-5.5
B	-5.3
A	-5.3
A	-5.3

[1] modes: A - the ones with PZQ aromatic part imbedded within the β-cyclodextrin cavity (Figure 5a in the manuscript), B - the ones with cyclohexane and central pyrazino ring of PZQ within the β-CD cavity (Figure 5b in the manuscript), C - in-between modes A and B.

7. Linearity of UPLC-DAD method used for identification of the degradation products

Table S6. Linearity of UPLC-DAD method, concentration range 0.1 – 2.5 mg mL^{-1})

	Linearity	R^2
PZQ	y = 7109.46x + 33.572	0.9968
PZQ-HPβCD	y = 2105.84x + 35.944	0.9992
PZQ-RMβCD	y = 1798.45x + 18.986	0.9995
PZQ-SBEβCD	y = 994.755x + 7.075	0.9998

8. Stress conditions

Table S7. List of detected degradations products for PZQ and PZQ/CD complexes formed under stress conditions (1M HCl, 1M NaOH, 3% H_2O_2)

	**HCl**	**NaOH**	**H_2O_2**	**Known/**
		observed *m/z*		**unknown**
**PZQ**	203, 331	331	329, 345	known
			148, 217	unknown
**PZQ/CD**	203, 331	331	329, 345	known
**complexes**			148, 215, 217	unknown

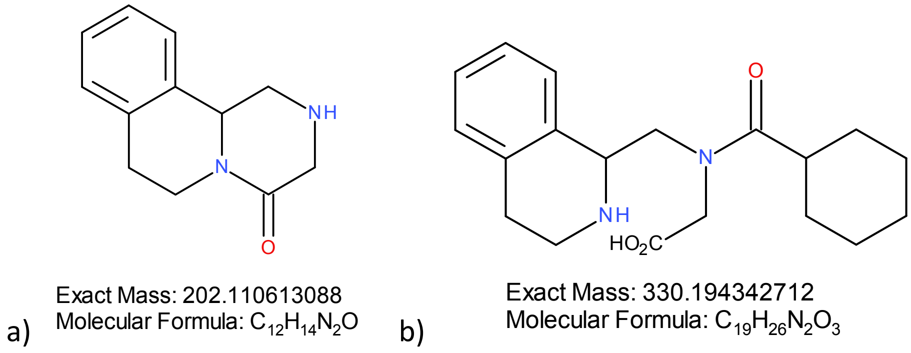

Figure S20. Structures of known degradants, a) *m/z* 203, b) *m/z* 331.

9. Accelerated solid stress testing

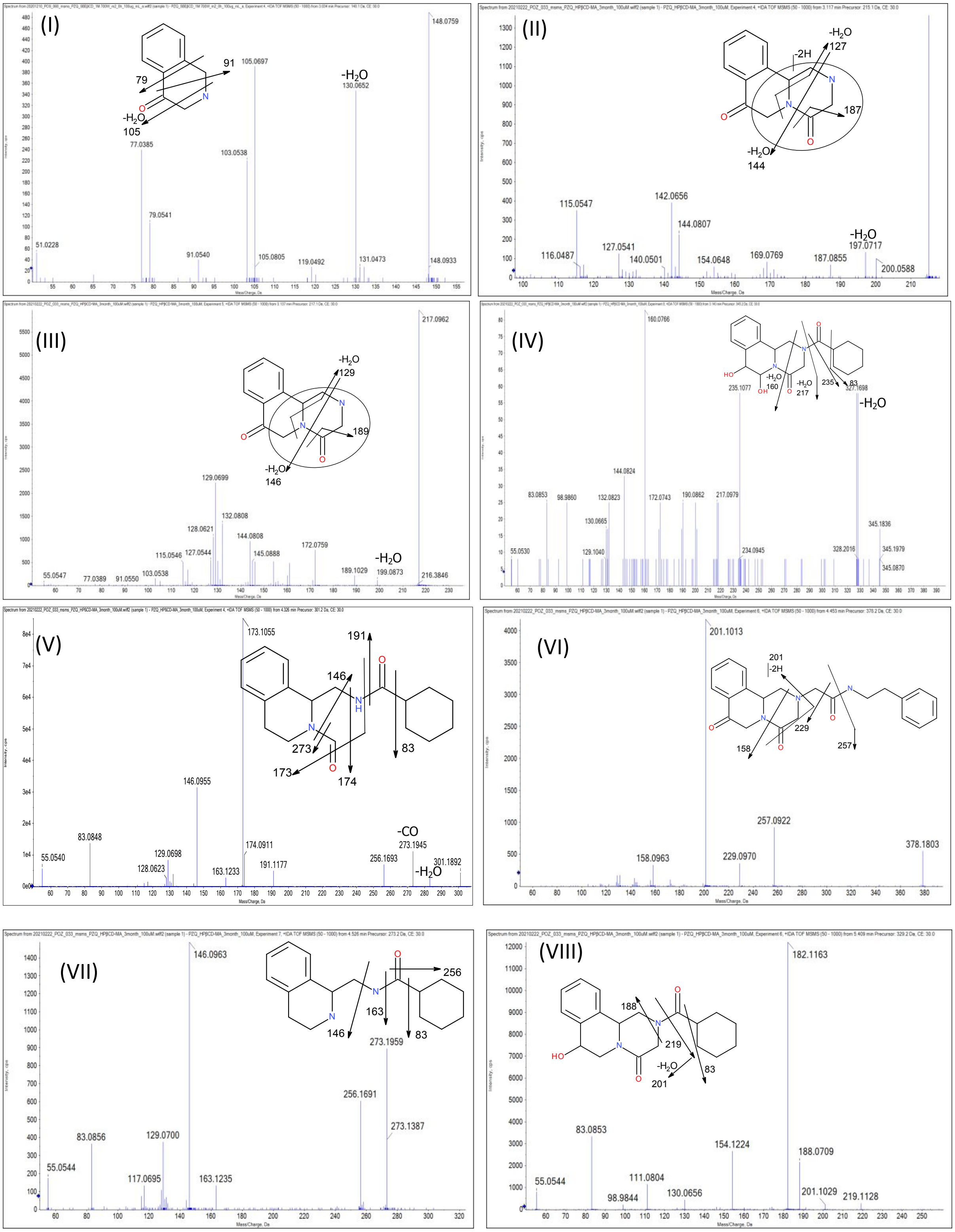

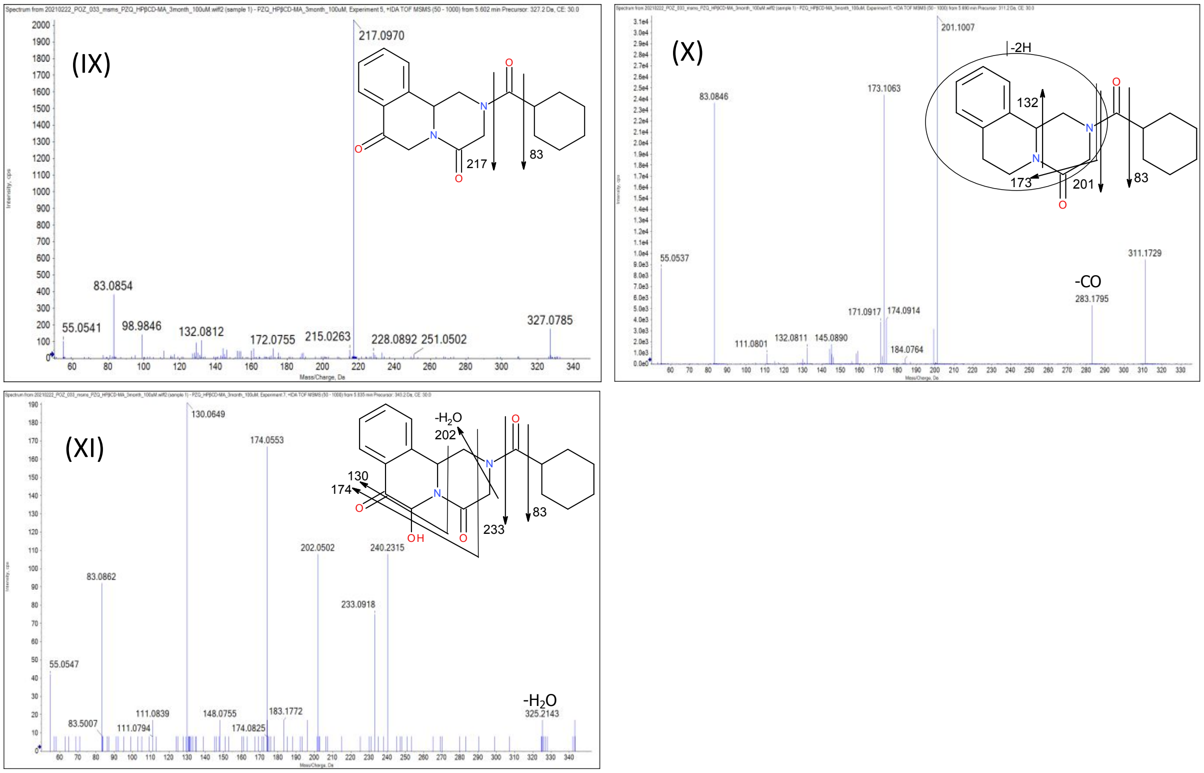

Figure S21. Tandem MS spectra and proposed chemical structures of detected degradation products from accelerated solid stress (3 months at 40 °C and 75% RH); I (RRT 0.57), II (RRT 0.57), III (RRT 0.60), IV (RRT 0.77), V (RRT 0.86), VI (RRT 0.90), VII (RRT 0.90), VIII (RRT 1.10), IX (RRT 1.14), X (RRT 1.17) and XI (RRT 1.23).

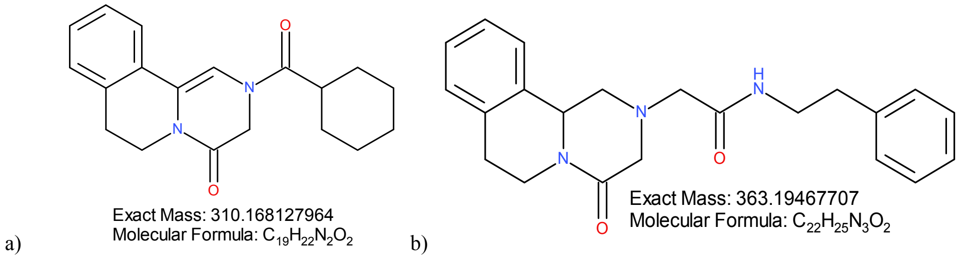

Figure S22. Structure of known PZQ impurities, a) MW310, b) MW 363.

References

1. Pretsch, Ernö, Bühlmann, Philippe, Badertscher M (2000) Structure Determination of Organic Compounds, 3rd ed. Springer Verlag, Berlin

2. Rymdén R.; Carlfors J.; Stilbs P. Substrate binding to cyclodextrins in aqueous solution: A multicomponent self-diffusion study. *J. Incl. Phenom*. **1983**, 1, 159–167 . https://doi.org/10.1007/BF00656818

3. Lin M.; Jayawickrama D.A.; Rose R.A.; DelViscio J.A.; Larive C.K.; Nuclear magnetic resonance spectroscopic analysis of the selective complexation of the cis and trans isomers of phenylalanylproline by β-cyclodextrin. *Anal. Chim. Acta* **1995**, 307, 449–457 . https://doi.org/10.1016/0003-2670(95)00006-L

4. Calderini A.; Pessine F.B.T.; Synthesis and characterization of inclusion complex of the vasodilator drug minoxidil with β-cyclodextrin. *J. Incl. Phenom. Macrocycl. Chem*. **2008**, 60, 369–377 . https://doi.org/10.1007/s10847-007-9387-z
